# Monitoring Changes in Sperm Concentration and Quality Help Improve Captive Breeding Outcomes of the Endangered Amphibian *Rawlinsonia littlejohni*

**DOI:** 10.1002/jez.70136

**Published:** 2026-10-05

**Authors:** Nadine Nolan, Anne Ibbotson, Natalie Calatayud, Matt W. Hayward, Alex Callen, Kaya Klop‐Toker, Rose Upton

**Affiliations:** ^1^ Amphibian Integrated Conservation Unit (Amphibian ICU) of the Center for Conservation Science, School of Science University of Newcastle Callaghan Australia; ^2^ Museums Victoria Research Institute Victoria Australia; ^3^ Center for African Conservation Ecology Nelson Mandela University Gqeberha South Africa

**Keywords:** assisted reproductive technologies, biobanking, breeding programs, captive insurance program, hormonal induction, reproductive cycles, sperm release

## Abstract

Amphibians are among the most threatened taxonomic groups, with many species experiencing rapid declines that have resulted in small, isolated populations vulnerable to inbreeding depression and reduced fertility. One major outcome of inbreeding is lowered fecundity, often linked to morphological changes in gametes. Littlejohn's tree frog (*Rawlinsonia littlejohni*; previously *Litoria*) is a threatened species requiring targeted conservation management. To increase population numbers and restore genetic diversity, a captive breeding program has been established. This study aimed to optimize the timing of hormonally‐induced sperm collection to improve outcomes for captive breeding, cryopreservation, and in vitro fertilization. Three experiments were conducted with ten captive males. Experiment one examined the effects of different human Chorionic Gonadotropin (hCG) and Gonadotropin‐releasing hormone antagonist (GnRH‐a) concentrations on spermiation response over 6 h. Both 5 and 10 IU hCG/g bwt yielded high sperm concentrations with similar motility and membrane viability, whereas GnRH‐a (1 and 2 µg/g bwt) produced no response. Experiment two assessed seasonal effects on sperm traits over a year, showing high variability between individuals but no consistent seasonal pattern. Sperm motility and membrane viability remained high, with slight declines in January, April, and June. Experiment three compared captive and wild males during the breeding season. Sperm concentration was higher in June for wild frogs but variable across all months in both groups. Our findings demonstrate that sperm production and quality in *R. littlejohni* are shaped more by its breeding system than by seasonal cycles, informing strategies to enhance reproductive success and genetic recovery in captivity.

## Research Highlights

- Optimized hCG dosing improved sperm yield and quality in *Rawlinsonia littlejohni*, while GnRH‐a was ineffective. Sperm traits were consistent year‐round.

## Graphical abstract

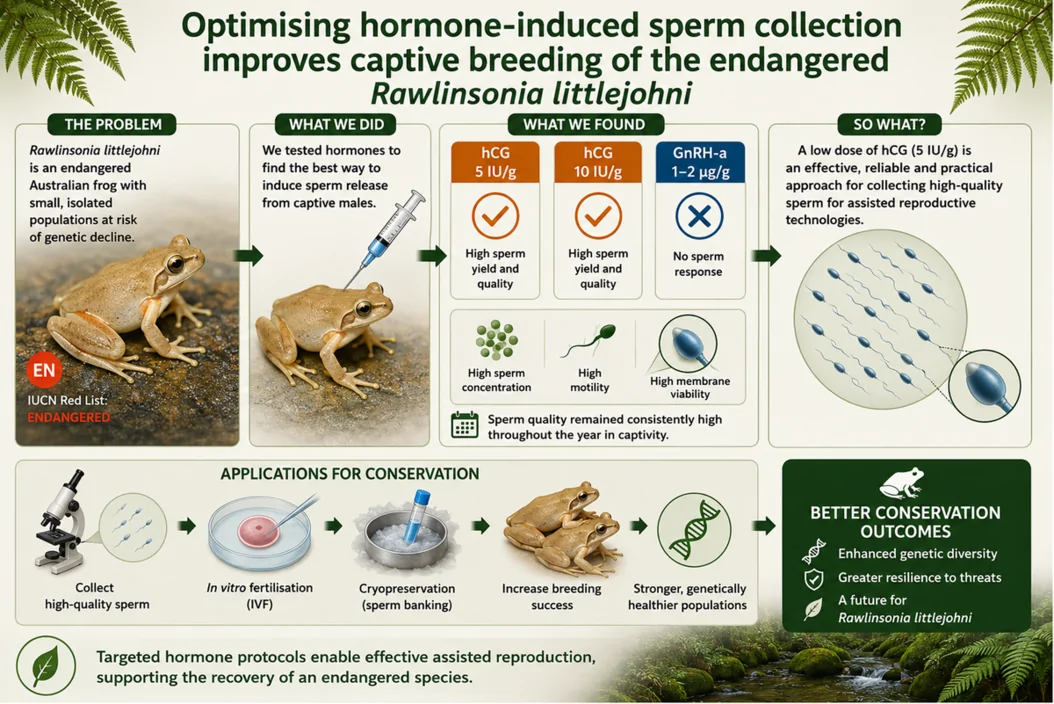

## 1 Introduction

Amphibians are the most threatened vertebrate taxa globally (IUCN 2023), largely due to habitat loss and degradation, invasive species, and the global spread of an invasive fungus that causes the disease chytridiomycosis (Blaustein et al. 2018; IUCN 2024; Luedtke et al. 2023; Scheele et al. 2019). Threats to amphibians have been further exacerbated by climate change (Cayuela et al. 2016; Nolan et al. 2023). These threats have resulted in many amphibian species existing in small, isolated populations, which, coupled with their reduced dispersal ability, results in genetic isolation (Rowan et al. 2022; Stock et al. 2023; Wilk et al. 2020). These populations are at greater risk of inbreeding depression, leaving them vulnerable to extinction from stochastic events, such as drought (Frankham et al. 2019; Hinkson and Poo 2020). Inbreeding depression arises when mating occurs between closely related individuals, increasing the likelihood of offspring inheriting deleterious recessive alleles (Charlesworth and Charlesworth 1999). Given the elevated extinction risk amphibians currently face, *ex situ* conservation actions, such as captive breeding programs, need to maintain or improve the reproductive fitness of individuals to maximize conservation outcomes and bring species back from the brink.

Captive breeding programs provide a means to secure threatened amphibian populations while maintaining genetic diversity and producing individuals suitable for reintroduction or translocation initiatives (Griffiths and Pavajeau 2008; Beauclerc et al. 2010; Harding et al. 2016; Karlsdóttir et al. 2021). Captive breeding of amphibians often requires extensive knowledge of a species' natural history and reproductive biology, and breeding programs frequently experience suboptimal reproduction where gaps in that knowledge exist (Browne and Zippel 2007; Kouba et al. 2009; Karlsdóttir et al. 2021). Low fecundity creates a knock‐on effect that hinders the contribution of *ex‐situ* institutions to assist species recovery, as insufficient individuals are available for reintroductions and translocations (Della Togna et al. 2020; Julien et al. 2023). To bolster low reproductive outputs and to better regulate breeding events, assisted reproductive technologies (ARTs) such as hormone therapy and in vitro fertilization (IVF) can be implemented (Bergers and Hao Li 1960; Wolf and Hedrick 1971; Browne et al. 2006; Kouba et al. 2009). Cryopreserved sperm, combined with the use of hormonal gamete induction for IVF, can further enhance the contributions of *ex‐situ* conservation in allowing breeding programs to indefinitely store reproductive material, while optimizing the timing of reproductive procedures and improving control over gamete selection (Browne et al. 1998; Kouba et al. 2009; Kouba et al. 2013; Della Togna et al. 2020). Consequently, this approach reduces costs by minimizing the number of live adult frogs required in a colony whilst maintaining the genetic diversity of the founder animals and ensuring the production of genetically diverse individuals for potential reintroduction in the future (Kouba et al. 2013; Della Togna et al. 2020; Howell et al. 2021).

Captive breeding programs have been established for multiple threatened amphibian species, but their success has been varied, possibly because of a lack of understanding of reproductive cycles and the influence of exogenous and endogenous factors (Calatayud et al. 2022; Jorgensen 1992; Morrison and Hero 2003). For example, some amphibians exhibit seasonal reproductive patterns, where behavioral and physiological changes lead to intense mating efforts (Arregui et al. 2020; Duellman and Trueb 1994), while others display nonseasonal reproductive patterns, allowing them to reproduce year‐round in response to specific and variable environmental cues (Whittier and Crews 1987). In seasonal breeding amphibians, testicular cycles can range from continuous sperm production to discontinuous, where there is a complete arrest of spermatogenesis during part of the year (Jorgensen 1992; Lofts 1974; Rastogi 1976). Exogenous factors affect and regulate the reproductive cycle of many amphibians and can include environmental (temperature, photoperiod, food availability) and social factors (behavioral interaction, experience) (Lukač et al. 2024; Rastogi et al. 2011). Endogenous factors also play an important role in reproductive cycles, with hormones and circannual rhythmicity regulating testicular and ovarian cycles in concert with exogenous factors (Clulow et al. 2018; Moore et al. 2005; Rastogi et al. 2011). Knowledge of reproductive cycles and the endogenous and exogenous factors that influence them are important when establishing successful captive breeding programs (Calatayud et al. 2018; Julien et al. 2023). This knowledge is particularly important for optimizing the timing of natural breeding or hormonal gamete induction for IVF or cryopreservation procedures, as selecting the best quality gametes or pairing reproductively mature individuals improves fecundity outcomes (Arregui et al. 2020; Arregui et al. 2022; Graham and Kouba 2022).

The endangered Littlejohn's tree frog, *Rawlinsonia littlejohni*, which inhabits the east coast of New South Wales, Australia, is in urgent need of conservation with only five small, isolated populations remaining (Stock et al. 2023). Genetic screening has revealed low genetic diversity and high inbreeding within remaining populations (Stock et al. 2023; Upton et al. 2024b). To address these issues, we established a captive breeding program to restore genetic diversity through outbreeding and crossbreeding of individuals from different populations. Understanding male reproductive cycles is critical for optimizing captive breeding outcomes and improving fecundity. While *R. littlejohni* typically breeds during winter (June to August), breeding has also been observed outside this period under optimal environmental conditions, like prolonged rainfall (Wallace 2023). This study aimed to optimize exogenous hormone administration for sperm collection, assess monthly sperm quality by measuring sperm concentration, motility and membrane integrity across a year, and compare sperm parameters between a captive and wild population to determine whether a mismatch between breeding phenology and sperm production parameters could explain the low fecundity of the species.

## 2 Methods

### 2.1 Study Species and Husbandry

Male *R. littlejohni* used in this experiment were collected from wild populations within the Central Coast Ranges, NSW, and housed at the University of Newcastle captive breeding facility. The Central Coast Ranges is comprised of two areas, Watagans National Park and Olney State Forest. These areas are situated at elevations above 100 meters, feature a mix of dry sclerophyll eucalypt forests on sedimentary formations on Narrabeen Shales and Hawkesbury Sandstone. Since the animals were originally sourced from wild populations, their exact age was unknown. However, males typically reach sexual maturity around 1 year of age, and all individuals exhibited secondary sexual characteristics, such as darkened nuptial pads, indicating sexual maturity. The weight of the males used in the study ranged from 10.7 ± 1.9 g (mean ± standard deviation, *n* = 10).

Individuals were kept in clear plastic tanks (18 × 30 x 20 cm) for the duration of the experiment (January 2023–March 2024). Each tank included a small container (6 × 11 x 9.5 cm) filled with 10% Simplified Amphibian Ringer (100% SAR; 113 mM NaCl, 1 mM CaCl_2_, 2 mM KCl, 3.6 mM NaHCO_3_; ~220 mOsm/kg; recipe) (Browne et al. 1998) solution and aged tap water, autoclaved leaf litter, and an artificial plant provided as a hide. The laboratory temperature was maintained at 22°C (± 2°), with UVB lighting on a 12:12‐h day‐night cycle. The tanks were monitored daily to check frog health, food availability, and water levels. Frogs were fed a diet of crickets, supplemented with calcium and vitamin powder (Multical Dust; Vetafarm), three times a week. Prior to the commencement of the experiment, all frogs were prophylactically treated with voriconazole, to clear any infection from chytrid fungus. Each frog was microchipped with an 8 mm passive integrated transponder (PIT tag) for individual identification, and webbing tissue was collected for genetic screening. All experiments were conducted in accordance with international, national, and institutional standards for the care and welfare of animals. Animals were housed under University of Newcastle ethics approval A‐2021‐119, while experiments were conducted according to A‐2022‐207, with initial collection of captive animals under NSW Scientific License SL102105 and SL102642 and reproductive work in wild populations under SL101269.

### 2.2 Sperm Collection and Assessment

For all experiments, hormones were administered subcutaneously via the dorsal lymph sac (see below for doses in specific experiments). Males were weighed to the nearest 0.1 g for the determination of hormone concentration to be administered by body weight. All injections were given using a 0.3 ml syringe and 31‐gauge needle. After frogs were injected with the designated hormone, they were placed in small plastic containers (5 cm D x 15 cm L x 9 cm W) filled with aged tap water to a depth of approximately 1 cm to promote urine production. Average volume of spermic urine collected per sampling event was approximately 494 µL (*n* = 166 samples). Spermic urine collection was obtained by inserting a thin plastic gel‐loading tip into the cloaca (Corning, New York, United States).

Urine samples were assessed for the presence of sperm at 400× magnification. Membrane integrity was determined using an Eosin‐Y dye exclusion assay, measured as the proportion of cells with intact plasma membranes. A 1:1 ratio of sperm sample to 0.5% wt/vol Eosin‐Yellow solution, diluted in 0.9% wt/vol saline (Upton et al. 2021), was mixed to achieve a total volume of 2 µL. The mixture was allowed to stand for a minimum of 30 s at room temperature before being observed under a coverslip at 400x magnification using brightfield illumination on a Nikon Eclipse E200. Spermatozoa that stained pink were considered compromised, as the dye permeated their cell membranes, while those with clear cytoplasm, indicating functioning cells that excluded the dye, were categorized as having intact membranes (Figure 1). To evaluate sperm motility, spermatozoa were counted and classified as (1) non‐motile, (2) motile without forward progression, or (3) motile with forward progression at 200x magnification using a Kyowa Unilux‐12 microscope and phase contrast optics. For both membrane integrity and motility assessments, two independent counts of at least 100 spermatozoa each were conducted across multiple fields of view, resulting in a minimum of 200 spermatozoa assessed per sample. Each assessment was prepared on a new slide.

**Figure 1:**
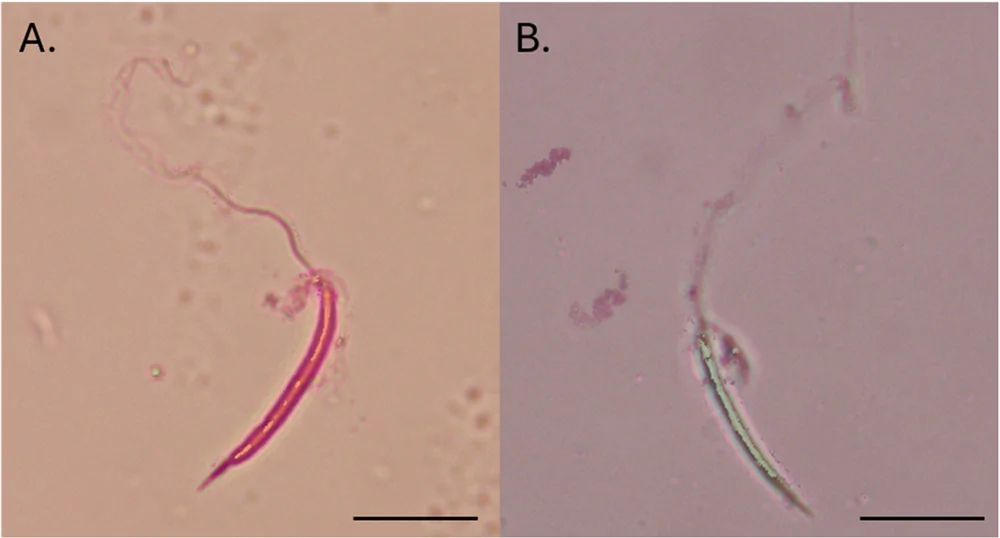
Eosin‐Y staining of *Rawlinsonia littlejohni* sperm under oil immersion. A) Pink‐stained cell indicates cell membrane damage and is scored as not intact; B) Clear cytoplasm indicates dye has been excluded and cell is counted as membrane intact. Scale bar equals 10 µm.

Sperm concentration was measured using a Neubauer improved haemocytometer counting chamber. A dilution with SAR was made to yield a sperm solution with immotile sperm (due to increase in osmolality) and which was not over concentrated (so that individual sperm were visible on the grid). If the spermic urine was dilute, then a raw sample were used, otherwise a 1:9 dilution was used. Ten microliters of the sperm solution were pipetted into each of two chambers, and sperm in at least 5 secondary quadrats per chamber were counted to calculate the total sperm cells per milliliter. Where the spermic urine was more dilute, 25 secondary quadrats, or 4‐9 primary quadrats were counted to target > 50 sperm counted. The dilution factor and the number of primary or secondary quadrats counted were considered in the final calculation (Upton et al. 2024a).

### 2.3 Experiment One: Effect of hCG and GnRH‐a Concentrations on Spermiation Response of *R. littlejohni*

The effect of hCG (Chorulon, Intervet, Boxmeer, Netherlands) and GnRH‐a (des‐Gly^10^, d‐Ala^6^, Pro‐LHRH, Bachem, Bubendorf, Switzerland) concentrations on *R. littlejohni* spermiation in five males was tested under the following experimental treatments: (1) 10 IU/g body weight (bwt) hCG (10‐hCG); (2) 5 IU/g body weight hCG (5‐hCG); (3) 1 µg/g body weight GnRH‐a (1‐GnRH); (4) 2 µg/g body weight GnRH‐a (2‐GnRH); and (5) no hormone (control—200 µL of Simplified Amphibian Ringer, SAR). All hormone treatments were made up to a total of 200 µl with SAR. These hormone concentrations were selected due to their widespread use in inducing hormonal responses in closely related species in previous studies (Silla et al. 2019; Upton et al. 2024a) and in an attempt to refine previous doses to below 20 IU/g bwt. Spermic urine was collected every hour for 6 h post‐injection and assessed for concentration, motility, and membrane integrity. A urine sample was collected from all frogs prior to hormone injection (hour zero) and all frogs were found to be aspermic.

Due to the limited number of reproductively mature male *R. littlejohni* available in the captive colony, this experiment was conducted in two phases using a repeated‐measures crossover design, in which individuals were exposed to multiple treatments across several cycles (Figure 2). In the first treatment cycle each of the five males (Frogs A–E) were randomly assigned a hormone dose (10 IU or 5 IU of hCG and 1 µg or 2 µg of GnRH‐a) and a control dose (SAR). The four hormone‐treated frogs (two assigned to hCG treatments and two to GnRH‐a treatments) received a single hormone treatment per cycle and were sequentially rotated through the different hormone doses across treatment cycles, with each cycle separated by a 3‐week period. This time frame is consistent with intervals used in similar studies (Delgado et al. 1989; Haczkiewicz et al. 2017; Rastogi et al. 2011; Segatelli et al. 2009). The control frog (Frog E; *n* = 1) remained the same across phase 1. To enhance statistical robustness and power, the experiment was conducted in two phases using the same experimental design: the first from January to March 2023, and the second from November 2023 to February 2024. In phase 2, five new frogs (F–J) with no prior exposure to exogenous hormonal induction were introduced, along with two individuals from the first phase. Frog E, which served as the control in phase 1, was subsequently included in the hCG treatment cycles, while Frog D, which had received hormone treatments in phase 1, was reassigned as the control in phase 2. This crossover design increased the number of unique individuals contributing to each hormone treatment and dose, with ten frogs contributing to each hCG treatment group across both phases, although individuals were measured repeatedly across cycles (Figure 2). The experimental design follows a crossover (Latin square‐type) framework, which is commonly used to maximize statistical power while reducing the number of animals required (Festing and Altman 2002; Pham and Brannelly 2022). To account for repeated measurements arising from the use of the same individuals across treatment cycles, frog ID was included as a random effect in the statistical modeling (outlined below). Given the lack of response observed to both doses of GnRH‐a in phase 1, these treatments were not repeated in phase 2. As a result, a total of four frogs contributed to each GnRH‐a dose across the study.

**Figure 2:**
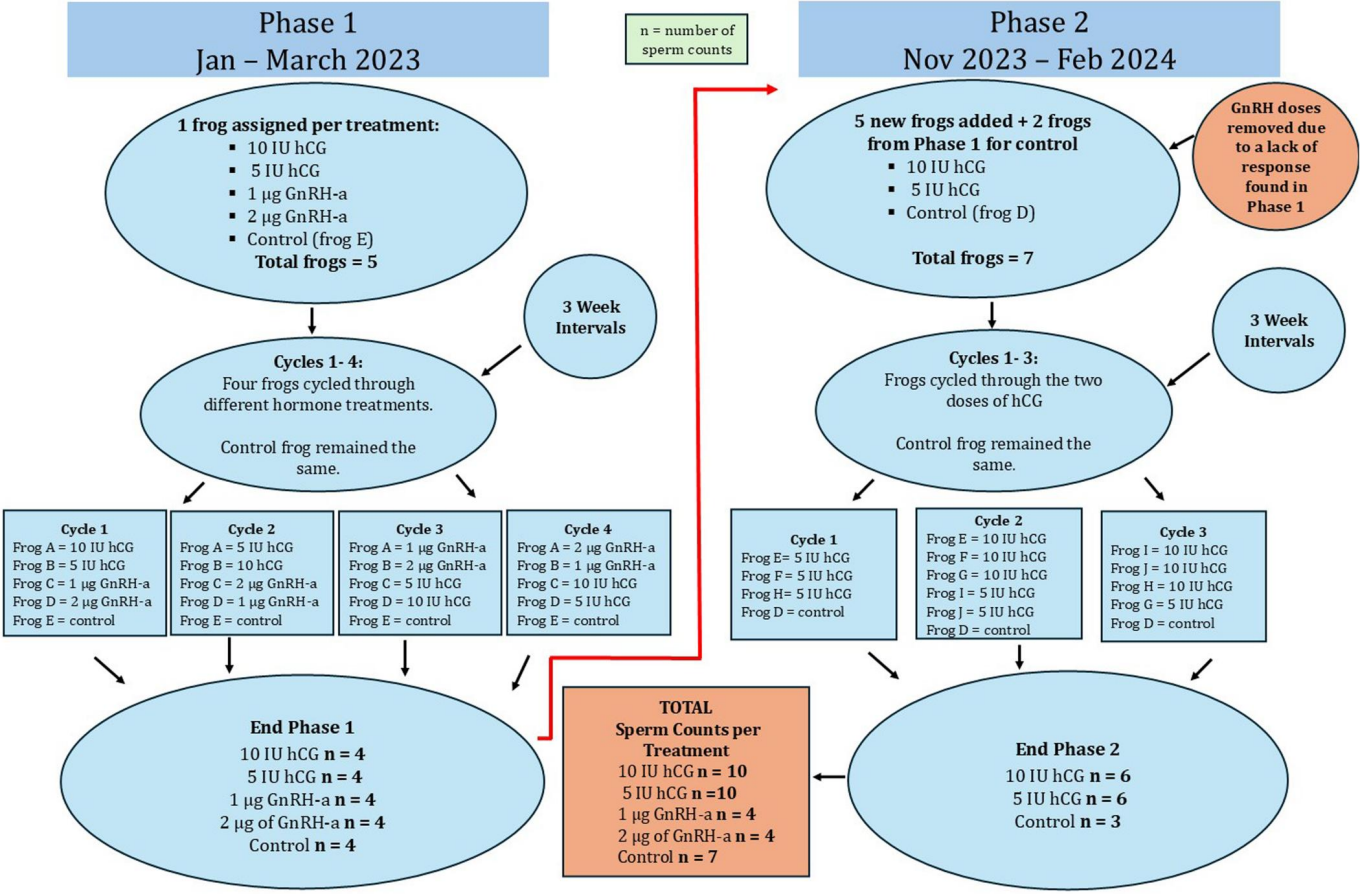
Experimental design for assessing the effects of hCG and GnRH‐a on sperm production in *Rawlinsonia littlejohni* over two phases.

### 2.4 Experiment Two: Determining the Effect of Month on Captive Animal Sperm Concentration and Quality

To test the effects of seasonality on *R. littlejohni* sperm quality, hormonally induced spermic urine were collected monthly from 10 males across a year (January–December 2023) using 5 IU/g btw hCG made up to 200 μL with SAR. The 5 IU/g bwt dose was selected based on the results of Experiment one, where it was found to be the lowest dose able to induce the desired sperm response. Spermic urine was collected every hour for 6 h post‐injection, as per Upton et al. (2024a). The samples from all 6 h were combined and assessed for total concentration, motility, and membrane integrity. Month eight (August) only contained four males with the other males being entered into the 2023 breeding tanks for that month. No breeding occurred and males were returned to experimental treatments in September.

### 2.5 Experiment Three: Differences in Sperm Concentration and Quality Between Wild and Captive Populations

To determine differences in sperm quality between wild and captive *R. littlejohni* populations, hormonally induced (5 IU/g bwt hCG) spermic urine was collected from a nearby wild population (Central Coast Ranges) across 4 months (June, July, August, September) during the species peak breeding season. Sperm concentration, motility and membrane integrity in the wild population were compared to the captive colony individuals assessed in Experiment two across the same months. The number of frogs used to assess wild sperm concentration and quality varied each month, depending on the number of males caught through visual encounter surveys. In June, 11 individuals were assessed; in July 10; in August 4; and in September 3 individual frogs were included.

Spermic urine collection and assessment for this experiment followed the same procedures as in the other two experiments, with the exception of the collection intervals and duration. Due to logistical constraints in the field, sperm assessments in the wild population were conducted 30 min after the injection and collected every 10 min over a 1‐h period. The collected samples were then combined to determine total concentration, motility, and membrane viability. Samples from the field were being collected for the purpose of biobanking genetic diversity of the population and were opportunistically included in this study. While the differences in collection times and intervals could lead to variability in results, the results of Experiment one show minimal biological differences in sperm concentration and quality measures over the 6‐h period with decreases observed mostly at hour six, and were thus considered a valuable comparison, despite the differences in method.

### 2.6 Statistical Analyzes

In all three experiments, four separate Generalized Linear Mixed Models (GLMMs) were used to analyze each of the sperm parameters, including concentration, total motility (the sum of non‐progressive and progressive sperm), forward progressive motility and membrane integrity. Each sperm parameter served as the dependent variable in its respective model. For sperm concentration, the raw counts were used with an offset of the log reciprocal of an adjustment factor (based on dilution factor, volume and the number of quadrats counted as appropriate) to convert the raw counts to cells/mL (Upton et al. 2024a). Based on the AIC as a model fit criterion, it was determined that all models for sperm concentration needed to use the negative binomial distribution to account for overdispersion. For the forward progressive motility, total motility, and membrane integrity models, which used proportion data, a binomial distribution with a logit link function was applied. Significance was determined by pairwise comparisons to determine odds ratios of expected counts between conditions and 95% confidence intervals (Upton et al. 2024a).

In Experiment one, given the lack of response to hormonal sperm induction from GnRH‐a, the final models used for each sperm parameter included only the two doses of hCG. Fixed effects included hormone concentration (10 IU/g bwt hCG, and 5 IU/g bwt hCG), hour since injection (ranging from 1 to 6 h). Random effects included frog ID, body condition (weight/snout‐vent‐length), count number (with two counts conducted per sperm sample), and treatment cycle (ranging from 1 to 4). Four separate models were run, testing the fixed effects against sperm concentration, total motility, forward‐progressive motility and membrane integrity. The interaction between treatment and hour was tested for each model and was found to be significant for membrane integrity, total motility, and forward progressive motility models. The interaction between treatment and hour was not significant for sperm concentration and therefore dropped from the model.

In Experiment two, the fixed effect included month (January–December). Random effects included frog ID, body condition (weight/snout‐vent‐length), and count number (with two counts conducted per sperm sample).

In Experiment three, fixed effects included location type (wild vs captive) and month (peak breeding time: June, July, August, September). Random effects included frog ID, body condition (weight/snout‐vent length), and count number (with two counts conducted per sperm sample). Four separate models were run, testing the fixed effects against sperm concentration, total motility, forward‐progressive motility and membrane integrity. The interaction between month and location was tested for all models and was found to be significant for sperm concentration. The interaction between month and location was not significant for membrane integrity, total motility, and forward progressive motility and therefore dropped from the models.

All analyzes were completed using R (Version 4.4.1) with R packages *glmmTMB* used for binomial and negative binomial GLMM modeling (Brooks et al. 2017). The *emmeans* package was used to calculate estimated marginal means (EMMs) and their 95% confidence limits. These were back transformed to represent sperm concentrations (cells/mL), as well as proportions for motility and membrane integrity. Ratios (for sperm concentration data) and odds ratios (for proportion data) comparing treatments were also generated using the *emmeans* package (Lenth 2022). All graphs were created using the *ggplot2* and *gridExtra* packages (Auguie and Antonov 2017; Wickham and Wickham 2016).

## 3 Results

### 3.1 Experiment One: Effect of hCG and GnRH‐a Concentrations on Spermiation Response of *R. littlejohni*

No sperm cells were detected in urine samples collected in Control frogs that received 200 µl of SAR. Both doses of GnRH‐a had minimal impact on sperm release in *R. littlejohni* and thus, responses could not be modeled (Figure 3). Only a small number of sperm cells were detected during the first and second hours of the experiment, with even fewer in the third and fourth hours. No sperm cells were detected in urine after the fourth hour. Therefore, the following results are based on the hCG measures only.

**Figure 3:**
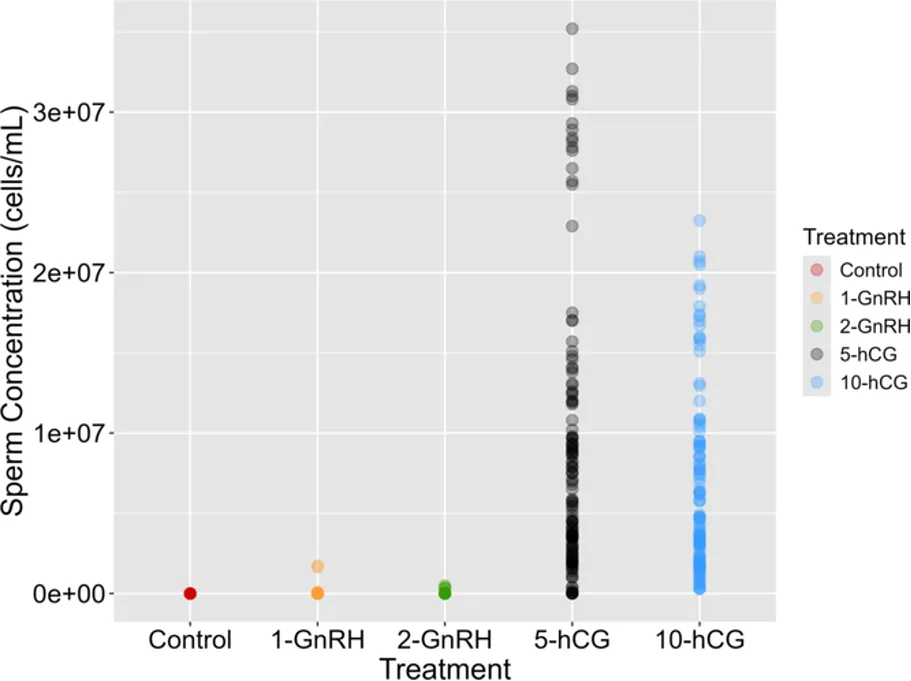
Raw hourly sperm concentration data (cells/mL) for each individual *Rawlinsonia littlejohni* following administration of two doses of GnRH‐a (1‐GnRH, *n* = 4; 2‐GnRH, *n* = 4) and hCG (5‐hCG, *n* = 10; 10‐hCG, *n* = 10), compared to the control group (*n* = 8). Data includes all hourly counts recorded for each individual over the sampling period.

There was a significant effect of collection hour (LRT χ^2^(5) = 56.85, *p* < 0.001, Figure 4A), but not hCG treatment (LRT χ^2^(1) = 0.610, *p* = 0.435), with decreasing concentration over time. Sperm concentration significantly decreased from 8.73 × 10^6^ sperm cells/mL (EMM, 95% CI: [5.18 × 10^6^, 1.47 ×10^7^]) at hour one to 2.56 × 10^6^ sperm cells/mL (EMM, 95% CI: [1.52 × 10^6^, 4.32 × 10^6^]) at hour six, with pairwise comparisons indicating that the expected sperm concentration at hour one was 3.4 times higher than at hour six (ratio = 3.4, 95% CI: 2.5, 4.7). Further pairwise comparisons demonstrated a decline in sperm concentration over time, especially between hours three to five and three to six, with fold differences (ratios of expected concentrations) ranging from 1.6 to 2.5 (see Supporting Information Table S1 for ratios and 95% CIs).

**Figure 4:**
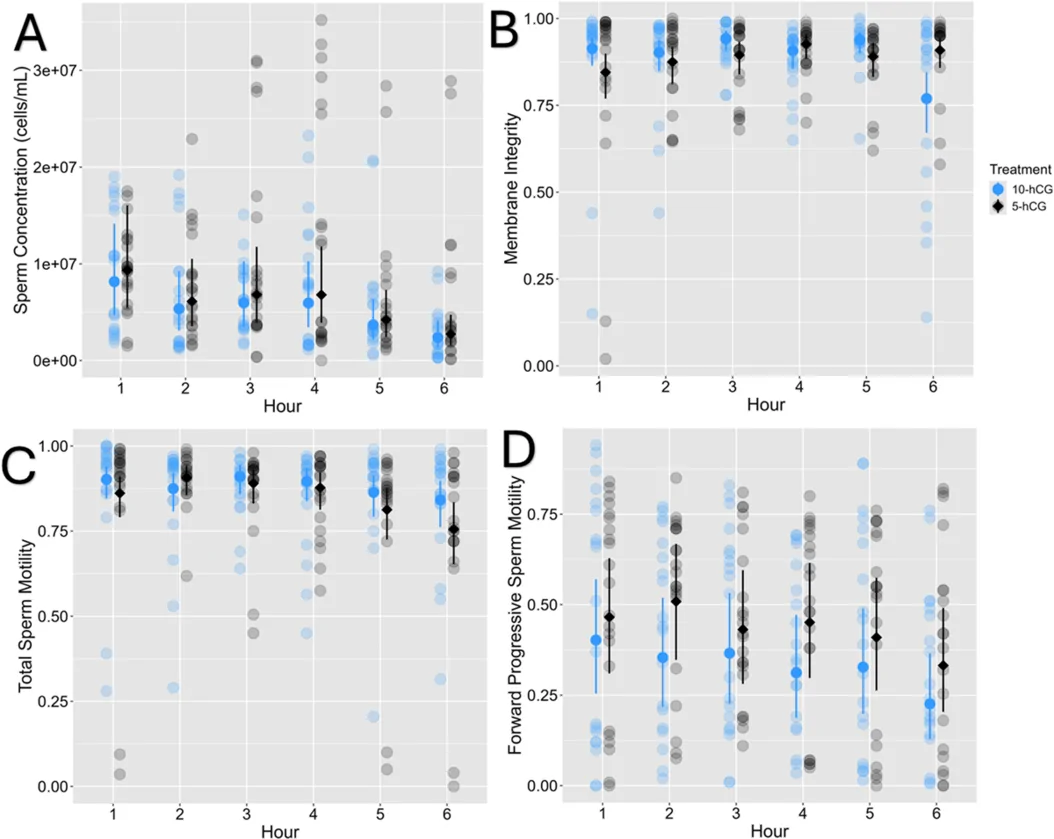
Raw sperm data and GLMM model means for the different hormone treatment groups (5‐hCG and 10‐hCG). A) Sperm concentration (cells/mL), B) Proportion of intact sperm membranes, C) Proportion total sperm motility, and D) Proportion forward progressive sperm motility. Solid diamonds represent estimated marginal means; Error bars reflect 95% confidence intervals and transparent points show raw data.

For membrane integrity, there was a significant effect of the interaction between treatment and collection hour (LRT χ^2^(3) = 129.09, *p* < 0.001). The proportion of cells with intact membranes for 5‐hCG had a 1.8‐fold increase from 84.5% at hour one (EMM, 95% CIs: [77, 89.9]) to 90.9% at hour 6 (EMM, 95% CI: [85.8, 94.3]) (OR: 1.83, 95% CI: [1.5,2.23]). The proportion of cells with intact membranes had a 1.6‐fold increase from 92.6% (EMM, 95% CI: [88.4, 95.4]) at hour 4 compared to 90.9% at hour 6 (EMM, 95% CIs: [85.8, 94.3]) (OR 1.62, 95% CI: [1.01, 1.58]; see Supporting Information Table S2 for full set of odds ratios and 95% CIs). For 10‐hCG, there was no clear peak in proportion of cells with intact membranes, but the highest was at hour three (EMM: 94.2%, 95% CI:[90.7, 96.5]; Figure 4B), see Supporting Information Table S2 for ratios and 95% CIs. Between the two treatments, the only timepoint where there was a significant difference was 6 h, where 5‐hCG had a threefold increase in predicted proportion of cells with intact membranes compared to 10‐hCG, with EMM of 90.9% (95% CI:[85.8, 94.3]) and 76.9% (95% CI:[67.1, 84.5]), respectively (OR: 2.98, 95% CIs: [1.8, 4.8]).

The interaction between treatment and collection hour had a significant effect on both total motility (LRT χ^2^(5) = 55.7, *p* < 0.001) and forward progressive sperm motility (LRT χ^2^(5) = 28.8, *p* < 0.001) (Figure 4C,D). There was a decrease in total and forward progressive motility over time, regardless of treatment. For 5‐hCG, there was up to a two‐fold increase in total motility at hour one (EMM: 86.1, 95% CI: [79.1, 91.1]) compared to hour five (EMM: 81.3, 95% CI: [72.5, 87.7]) and six (EMM: 75.5, 95% CI: [65.3, 83.5]) with odds ratio of 1.4 (CI: [1.21, 1.69]) and 2.01 (CI: [1.71, 2.36]) respectively. There was no clear peak in total motility for 10‐hCG, however there was a 1.7‐fold increase (OR 1.73, CI: [1.44, 2.08]) at hour one (EMM 90.2) compared to hour six (EMM 84.2). Forward progressive motility for 5‐hCG was not significantly different between hours one (EMM 46.6) and two (EMM 50.9) (OR 0.84, CI [0.74. 0.96]. However, forward motility at hour one was significantly higher than at hours three, five and six (ORs ranged from 1.15 to 1.75), with EMM at hour six decreasing to 33.2% [20.4, 49.1]. Additionally, forward progressive motility for 5‐hCG at hour two was significantly higher than at all other subsequent timepoints (ORs: 1.2 ‐ 2.1). For total motility and forward progressive motility there was no significant difference in timepoints between 5‐hCG and 10‐hCG (see Supporting Information Table S3 and S4).

### 3.2 Experiment Two: Determining the Effect of Month on Captive Animal Sperm Concentration and Quality

There was a significant effect of month on sperm concentration (LRT χ^2^(11) = 64.49, *p* < 0.0001; Figure 5A). Sperm concentration in October was significantly higher at 8.46 × 10^6^ sperm cells/mL (EMM, 95% CIs: [4.95 × 10^6^, 1.45 × 10^7^]) compared to January (EMM = 3.34 × 10^6^ cells/mL), February (EMM = 2.30 × 10^6^ cells/mL) and April (EMM = 1.40 × 10^6^ cells/mL), with pairwise comparisons showing ratios between 2.4 and 3.6. April had the lowest sperm concentration counts across all months at 1.40 × 10^6^ sperm cells/mL (EMM, 95% CIs: [7.46 × 10^6^, 2.64 × 10^6^]) and was significantly lower compared to May (EMM = 3.13 × 10^6^ cells/mL), August (EMM = 3.45 × 10^6^ cells/mL), September (EMM = 3.39 × 10^6^ cells/mL) and October (EMM = 8.46 × 10^6^ cells/mL)), with pairwise comparisons showing ratios between 2.2 and 6.02 (see Supporting Information Table S5 for ratios and 95% CIs).

**Figure 5:**
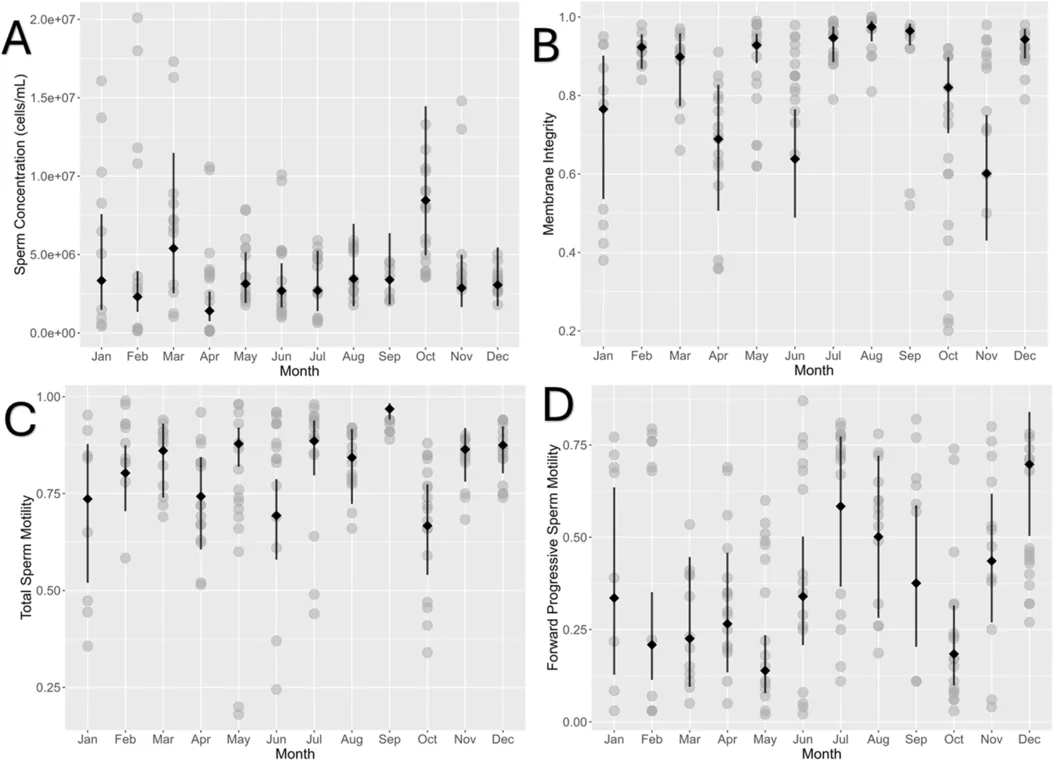
Raw sperm data and GLMM model means for different months. A) Sperm concentration. (cells/mL), B) Proportion of intact sperm membranes, C) Proportion total sperm motility, and D) Proportion forward progressive sperm motility. Solid diamonds represent estimated marginal means; Error bars reflect 95% confidence intervals and transparent points show raw data.

There was a significant effect of month on membrane integrity (LRT χ^2^(11) = 155.2, *p* < 0.0001; Figure 5B). There was a high predicted proportion of cells with intact membranes in February (EMM = 92.3), March (EMM = 89.8), May (EMM = 92.8), July (EMM = 94.7), August (EMM = 97.5), September (EMM = 96.50), and December (EMM = 94.3), with no significant differences between these months (see Supporting Information Table S5). The lowest proportion of intact membranes were observed in April (EMM = 68.9%, 95% CIs: [50.6, 82.7]), June (EMM = 63.8%, 95% CIs: [48.9, 76.5]), and November (EMM = 60.1%, 95% CIs: [43, 75]), with February, August and December having up to a 26‐fold increase compared to these months (Odds ratio ranges; February (April = 5.4 [2.05, 14.3], June = 6.8 [3.4, 13.7], and November = 7.9 [3.2, 19.8), August (April = 17.5 [5.2, 49.4], June = 22 [7.1,68.5], and November = 25.8 [9.98, 83.6]), and December (April = 7.5 [2.7, 20.6], June = 9.4 4.1, 21.6], and November = 10.9 [4.2, 28.8]), see Supporting Information Table S5 for ratios and 95% CIs.

There was a significant effect of the month on total sperm motility (LRT χ^2^(11) = 114.76, *p* < 0.0001; Figure 5C). September had the highest total motility at 96.8% (EMM, 95% CIs: [94.1, 98.3]) and was significantly higher compared to January (EMM = 73.6%), February (EMM = 80%), March (EMM = 86.1%), April (EMM = 74.3%) and May (EMM = 87.9%), with pairwise comparisons showing ratios ranging from 4.2 to 10.96. Odds ratios indicate that there is a 4 ‐ 10‐fold increase in that month's likelihood of having sperm with greater total motility (see Supporting Information Table S6 for ratios and 95% CIs).

There was a significant effect of the month on forward progressive sperm motility (LRT χ^2^(11) = 122.55, *p* < 0.0001; Figure 5D). December had the highest forward progressive sperm motility at 69.8% (EEM, 95% CIs: [50.3, 84]) and was significantly higher than all other months, except July (EMM = 58.4%) and August (EMM = 50.2%), with pairwise comparisons showing ratios ranging between 2.9 and 14.3 (see Supporting Information Table S6 for ratios and 95% CIs). Ratios indicated that there is a 3‐14 fold increase in December's likelihood of having sperm with greater forward progressive motility. May showed the lowest forward progressive motility at 13.9% (EMM, 95% CIs: [7.8,23.5]).

### 3.3 Experiment Three: Differences in Sperm Concentration and Quality Between Wild and Captive Populations

The interaction between month and location had a significant effect on sperm concentration (LRT χ^2^(3) = 17.21, *p* < 0.001; Figure 6A). In June, wild frogs had significantly higher sperm concentrations (EMM = 8.35 × 10^6^ sperm cells/mL, 95% CIs: [5.08 × 10^6^, 1.37 × 10^7^]) compared to captive frogs (EMM = 2.88 × 10^6^ sperm cells/mL, 95% CIs: [1.57 × 10^6^, 5.29 × 10^6^]), with a ratio of 2.89 [1.3, 6.3]. By August, the trend reversed, with wild frogs showing significantly lower sperm concentrations (EMM = 7.77 × 10^5^ sperm cells/mL, 95% CIs: [3.27 × 10^5^, 1.84 × 10^6^]) compared to captive frogs (EMM = 3.49 × 10^6^ sperm cells/mL, 95% CIs: [1.86 × 10^6^, 6.54 × 10^6^ 6]), with a ratio of 4.5 [1.5, 13.1] (see Supporting Information Table S7 for ratios and 95% CIs). No significant differences were observed in July or September between the wild and captive populations, and sperm concentration in captive frogs remained stable across all months.

**Figure 6:**
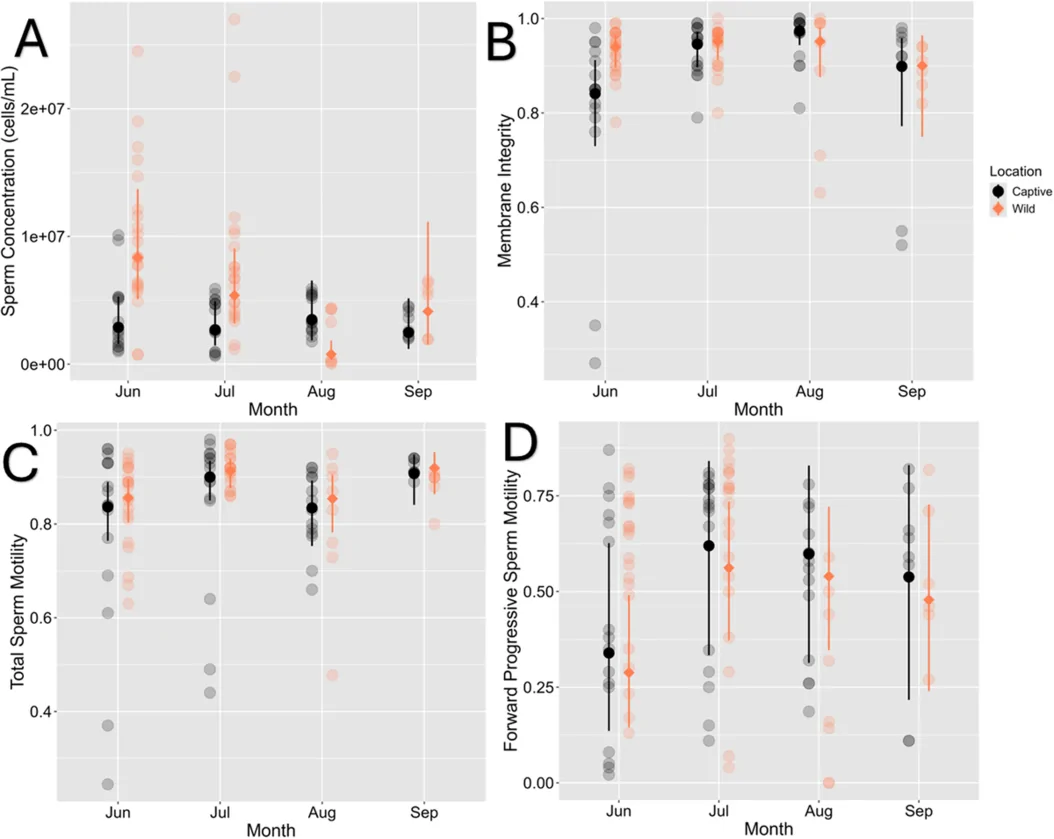
Raw sperm data and GLMM model means across different months between captive and wild individuals. A) Sperm concentration (cells/mL), B) Proportion of intact sperm membranes, C) Proportion total sperm motility, and D) Proportion forward progressive sperm motility. Solid diamonds represent estimated marginal means; Error bars reflect 95% confidence intervals and transparent points show raw data.

There was a significant effect of month (LRT χ^2^(3) = 10.32, *p* = 0.015), but not location (LRT χ^2^(1) = 1.06, *p* = 0.30) on membrane integrity. The proportion of cells with intact membranes was significantly higher in August (EMM = 97%, 95% CIs: [94.2, 98.5] compared to June (EMM = 90.6%, 95% CIs: [85.8, 93.9] and September (EMM = 90.4%, 95% CIs: [81.3, 95.3], with pairwise comparisons showing ratios of 3.3 [1.5,7.45] and 3.4 [1.3, 9.3], respectively (see Supporting Information Table S8 for ratios and 95% CIs). The month of July (EMM = 95%, 95% CIs: [92.1, 96.9] also had significantly higher proportion of cells with intact membranes than June, with pairwise comparisons showing a ratio of 1.97 [1.02, 3.78].

There was a significant effect of month (LRT χ^2^(3) = 8.96, *p* = 0.0297), but not location (LRT χ^2^(1) = 0.38, *p* = 0.534) on total sperm motility (Figure 6C), with sperm motility increasing in July (EMM = 90.8%, 95% CIs: [87.4, 93.4]) and September (EMM = 91.5%, 95% CIs: [86, 95]), in both captive and wild frogs. Pairwise comparisons showed significant ratios for July compared to June (1.75 [1.1, 2.76]) and August (1.81 [1.0,3.1]) and significant ratios for September compared to June (1.9 [1.0, 3.5]) and August (1.97 [1.0,3.86]), see Supporting Information Table S9 for ratios and 95% CIs. There was no significant effect of the month (LRT χ^2^(3) = 4.22, *p* = 0.23) or between wild and captive individuals (LRT χ^2^(1) = 0.034, *p* = 0.85) for forward progressive sperm motility (Figure 6D). Forward progressive motility stayed stable across each month with a range of means between 40.6% and 61.2%.

## 4 Discussion

This study presents the first successful induction of spermiation in *R. littlejohni*, marking a significant advance in understanding the reproductive biology of this threatened species. We demonstrate that effective sperm release can be achieved using lower doses of human Chorionic Gonadotropin (hCG) than typically applied in similar species, reducing hormone use while maintaining high sperm quality. This refinement improves the efficiency and welfare of assisted reproduction protocols and provides a foundation for future in vitro fertilization and cryopreservation efforts. Beyond optimizing hormone dosage, the study also contributes to broader knowledge of reproductive physiology in *R. littlejohni*, revealing consistent spermiation responses across captive and wild males.

We found minimal differences in sperm motility and concentration over a 12‐month period in captive individuals, as well as minimal differences in motility and membrane integrity between wild and captive populations. However, wild populations exhibited higher sperm concentrations in June, followed by a decline in subsequent months, suggesting greater variability across months in sperm concentration. Sperm concentration was generally lower in the captive population and remained constant across months. This indicates potential to improve captive conditions by more closely replicating environmental cues that stimulate spermatogenesis in the wild, such as fluctuations in temperature, photoperiod, and rainfall. Enhancing these environmental triggers within captive systems could promote more natural cycles of sperm production and improve reproductive output. As captive breeding programs become increasingly vital for conserving highly vulnerable amphibians, integrating such ecological understanding into husbandry practices is essential for optimizing conservation success.

There were no significant differences between the two tested doses of hCG, whereas hCG was more effective than GnRH‐a at inducing sperm release under the conditions tested. GnRH‐a elicited a minimal spermiation response, with six out of eight frogs releasing small amounts of sperm and only two individuals releasing sperm beyond the second hour (1–2 sperm cells/haemocytometer grid). These findings are consistent with previous studies showing reduced spermiation response to GnRH‐a relative to hCG in some p*elodryadid* frogs (Silla et al. 2019; Upton et al. 2024a). One possible explanation is that the GnRH‐a doses used here were not optimal for this species, as lower doses have been reported to improve responses in other frogs (Silla et al. 2019). Alternatively, responsiveness to GnRH‐a may vary among species. Further dose‐response studies would be needed to distinguish between these possibilities.

Our results support hCG as the more effective hormone for inducing spermiation in *R. littlejohni*, with no significant differences in sperm concentration, motility, or membrane integrity between the 5 IU/g bwt and 10 IU/g bwt doses. While some previous studies have found higher doses to be more effective in inducing high quality sperm (Clulow et al. 2018; Kouba et al. 2012; Silla and Roberts 2012), our results align with other results showing no significant difference between these two doses (Silla et al. 2019). Furthermore, our findings align with Clulow et al. (2018), who reported that lower doses of hCG (60 IU total, compared to ~ 50 and 100 IU in our study) in *Ranoidea chloris* yielded higher sperm concentrations compared to higher doses. This suggests that lower doses of hCG can be more effective for inducing spermiation in some amphibian species, including *R. littlejohni*. Overstimulation from higher doses may lead to adverse effects such as hormonal disruption or fluid regulation issues (Burgos and Vitale‐Calpe 1967; Uteshev et al. 2012; Ratner et al. 2014; Silla et al. 2021), making lower doses not only safer but also more cost‐effective for captive breeding programs, IVF, and cryopreservation efforts. While minimal biological differences in sperm parameters were observed across the six collection hours, recent studies have shown that there may be multiple peaks in sperm concentration and quality over a 72‐h period post hormone treatment (Calatayud et al. 2025; Otero et al. 2023). Given *R. littlejohni* pairs have been observed in amplexus for over 3 days (R. Upton, *unpubl. data*), there may be peaks in sperm quality not captured in the current study that could warrant further investigation. Together, these results suggest that a lower hCG dose may be sufficient for inducing spermiation in this species.

Our results did not reveal a clear seasonal peak in sperm concentration across the sampled month in captive male. This differs from species with more distinct seasonal reproductive patterns, where sperm traits are more closely aligned with the breeding season (Arregui et al. 2020). Although *R*. *littlejohni* has been observed breeding outside the peak winter period under favorable conditions, the present data are not sufficient to determine whether the species is truly continuous in its reproductive activity. Instead, our results suggest that sperm production in captivity may be relatively stable across the year. Membrane integrity varied among months, but without a clear seasonal pattern and even where significant differences were detected, these differences were not biologically meaningful. It is unclear what caused these variable results, however it is possible that fluctuations in membrane integrity may be linked to other environmental or physiological factors not measured in this study, such as changes in nutrition or stress levels, which could influence sperm quality independently of concentration or motility (Carr 2011; Guha et al. 1980; Keogh et al. 2018).

We observed substantial variation in sperm traits among individuals within the same month, with no consistent temporal trend. This individual‐level variability suggests that sperm quality should be assessed directly when selecting samples for assisted reproduction or biobanking, rather than inferred from sampling month alone. A practical implication of this result is that collecting and evaluating sperm from multiple males may be more informative than relying on a presumed seasonal peak.

Comparisons between captive and wild males should be interpreted cautiously because post‐injection collection schedules differed between field and captive sampling. However, given that Experiment one showed only modest changes in sperm traits across the first 6 h post‐injection, the comparison remains informative as an initial assessment. Captive conditions may buffer temporal variability in sperm production, whereas wild frogs are exposed to more variable environmental conditions. Further comparisons using consistent collection protocols would help clarify the extent to which the observed differences reflect methodology versus biology.

In addition, integrating standardized assessment frameworks such as the Wildlife Sperm Index (WSI) can enhance cross‐study comparability and improve decisions regarding gamete preservation. The WSI provides a composite score based on weighted sperm quality metrics, including motility, viability, and DNA integrity, allowing for quantitative, species‐specific evaluation of gametes (Jacobs et al. 2026). Incorporating such indices in future work with *R. littlejohni* could help determine which individuals contribute high‐quality samples suitable for biobanking and reduce the loss of viable genetic material due to inconsistent or subjective assessment. Further comparisons using consistent collection methodologies may provide further information on differences between wild and captive populations to further improve captive conditions. Our results suggest that changes, such as a reduction in temperature during winter months may improve sperm concentration under captive conditions. Additionally, controlled laboratory experiments could further elucidate which environmental variables contribute to changes in spermiation.

Our results broadly align with those of Julien et al. (2023), who reported higher sperm concentration in semi‐captive and wild individuals compared to fully captive males, while sperm quality metrics such as motility and membrane integrity remained similar between groups. In our study, wild males exhibited higher sperm concentrations than captive males during June, although this difference was not consistent across all months. This study attributed the lower sperm concentration in captive males to the absence of natural environmental cues, highlighting the role of the external environment in maintaining higher sperm production in more natural settings. Another contributing factor may be genetic diversity of the species. This species has significant inbreeding within its populations (Stock et al. 2023). Studies in other amphibians have found that this can lead to reduced fitness and sperm quality (Gage et al. 2006; Hinkson and Poo 2020; Mehlis et al. 2015). Given the severity of inbreeding in this species, this warrants further investigation.

## 5 Conclusion

Optimizing hormonal spermiation, including selecting appropriate hormone types and doses, is crucial for enhancing sperm concentration and quality, thereby improving the success of IVF and cryopreservation efforts in conservation initiatives. Our study demonstrated that hCG is the most effective hormone for inducing spermiation in *R. littlejohni*. Sperm parameters exhibited minimal variation across the six collection hours, indicating no clear optimal time for sperm collection. Instead, our findings suggest that a flexible approach to sperm collection may be necessary, given the high individual variability and lack of distinct temporal patterns. Wild males exhibited higher sperm concentrations than captive males during June, suggesting environmental factors may impact sperm variability. From the results of this study, we recommend using a low dose of 5 IU/g bwt hCG for this species, especially where repeated dosages may be needed over time. Further investigations are warranted to refine environmental and social cues within breeding facilities that promote natural amplexus events and produce high‐quality sperm and to investigate the role of genetics, diet and other factors. Increasing our understanding of reproductive physiology of a species, and the factors that are essential for establishing effective breeding programs can help rescue endangered amphibian species from extinction.

## Author Contributions

Conceptualization: Nadine Nolan, Anne Ibbotson, Rose Upton, Natalie Calatayud, Matt W. Hayward, Kaya Klop‐Toker, and Alex Callen. Data collection: Nadine Nolan, Anne Ibbotson, and Rose Upton. Statistical analysis: Nadine Nolan and Rose Upton. Writing – original draft preparation: Nadine Nolan. Writing – review and editing: Nadine Nolan, Anne Ibbotson, Rose Upton, Alex Callen, Kaya Klop‐Toker, and Matt W. Hayward. All authors have read and agreed to the published version of the article.

## Ethics Statement

Animal ethics approval came from the University of Newcastle (A‐2021–119 and A‐2022‐207) and work was conducted under scientific licence SL 102642.

## Conflicts of Interest

The authors declare no conflicts of interest.

## Supporting information


Supporting File

## Data Availability

The data that support the findings of this study are available from the corresponding author upon reasonable request.
