# Supplementary material for: Monitoring Changes in Sperm Concentration and Quality Help Improve Captive Breeding Outcomes of the Endangered Amphibian Rawlinsonia littlejohni
Source: J Exp Zool A Ecol Integr Physiol. 2026 Oct 5;345(10):1078–90. doi: 10.1002/jez.70136 (PMC13725229; doi:10.1002/jez.70136)
Supplement: Supplementary file 1 [file JEZ-345-1078-s001.docx]

**Monitoring changes in sperm concentration and quality help improve captive breeding outcomes of the endangered amphibian *Litoria littlejohni***

**Supplementary Materials:**

**Supplementary Table 1***: Pairwise Incidence Rate Ratios (IRRs) and 95% Confidence Intervals for Sperm Concentration in Litoria littlejohni, Comparing Different hCG Doses and Time Since Hormone Administration (n = 10 per treatment).*

| **Contrast** | **Ratio** | **LCL^1^** | **UCL^2^** | **Reverse Contrast** | **Ratio** | **LCL^1^** | **UCL^2^** |
| --- | --- | --- | --- | --- | --- | --- | --- |
| hour1 / hour2 | *1.52 | 1.12 | 2.08 | hour2 / hour1 | 0.66 | 0.48 | 0.89 |
| hour1 / hour3 | *1.37 | 1.00 | 1.87 | hour3 / hour1 | 0.73 | 0.53 | 1.00 |
| hour1 / hour4 | 1.37 | 0.99 | 1.90 | hour3 / hour2 | 1.12 | 0.82 | 1.52 |
| hour1 / hour5 | *2.21 | 1.61 | 3.05 | hour4 / hour1 | 0.73 | 0.53 | 1.01 |
| hour1 / hour6 | *3.41 | 2.47 | 4.71 | hour4 / hour2 | 1.11 | 0.81 | 1.52 |
| hour2 / hour3 | 0.90 | 0.66 | 1.22 | hour4 / hour3 | 1.00 | 0.73 | 1.36 |
| hour2 / hour4 | 0.90 | 0.66 | 1.23 | hour5 / hour1 | 0.45 | 0.33 | 0.62 |
| hour2 / hour5 | *1.45 | 1.06 | 1.98 | hour5 / hour2 | 0.69 | 0.50 | 0.94 |
| hour2 / hour6 | *2.24 | 1.63 | 3.07 | hour5 / hour3 | 0.62 | 0.45 | 0.84 |
| hour3 / hour4 | 1.00 | 0.74 | 1.37 | hour5 / hour4 | 0.62 | 0.45 | 0.85 |
| hour3 / hour5 | *1.62 | 1.19 | 2.21 | hour6 / hour1 | 0.29 | 0.21 | 0.41 |
| hour3 / hour6 | *2.49 | 1.82 | 3.41 | hour6 / hour2 | 0.45 | 0.33 | 0.61 |
| hour4 / hour5 | *1.61 | 1.18 | 2.21 | hour6 / hour3 | 0.40 | 0.29 | 0.55 |
| hour4 / hour6 | *2.48 | 1.81 | 3.41 | hour6 / hour4 | 0.40 | 0.29 | 0.55 |
| hour5 / hour6 | *1.54 | 1.13 | 2.10 | hour6 / hour5 | 0.65 | 0.48 | 0.88 |

^1^Lower confidence limit

^2^Upper confidence limit

- Significant pairwise comparisons

**Supplementary Table 2:** *Pairwise Odds Ratios (ORs) and 95% Confidence Intervals for Membrane Integrity in Litoria littlejohni, Comparing Different hCG Doses and Time Since Hormone Administration (n = 10 per treatment).*

| **Contrast** | **Ratio** | **LCL^1^** | **UCL^2^** | **Reverse Contrast** | **Ratio** | **LCL^1^** | **UCL^2^** |
| --- | --- | --- | --- | --- | --- | --- | --- |
| 10-hCG hour1 / 5-hCG hour1 | *1.93 | 1.19 | 3.14 | 5-hCG hour1 / 10-hCG hour1 | 0.52 | 0.32 | 0.84 |
| 10-hCG hour1 / 10-hCG hour2 | 1.14 | 0.93 | 1.39 | 10-hCG hour2 / 10-hCG hour1 | 0.88 | 0.72 | 1.07 |
| 10-hCG hour1 / 5-hCG hour2 | 1.51 | 0.93 | 2.45 | 10-hCG hour2 / 5-hCG hour1 | *1.70 | 1.05 | 2.76 |
| 10-hCG hour1 / 10-hCG hour3 | 0.65 | 0.52 | 0.81 | 5-hCG hour2 / 10-hCG hour1 | 0.66 | 0.41 | 1.08 |
| 10-hCG hour1 / 5-hCG hour3 | 1.24 | 0.76 | 2.02 | 5-hCG hour2 / 5-hCG hour1 | *1.28 | 1.08 | 1.52 |
| 10-hCG hour1 / 10-hCG hour4 | 1.08 | 0.88 | 1.32 | 5-hCG hour2 / 10-hCG hour2 | 0.75 | 0.46 | 1.22 |
| 10-hCG hour1 / 5-hCG hour4 | 0.84 | 0.51 | 1.38 | 10-hCG hour3 / 10-hCG hour1 | *1.55 | 1.23 | 1.94 |
| 10-hCG hour1 / 10-hCG hour5 | 0.70 | 0.56 | 0.88 | 10-hCG hour3 / 5-hCG hour1 | *2.99 | 1.83 | 4.90 |
| 10-hCG hour1 / 5-hCG hour5 | 1.30 | 0.79 | 2.12 | 10-hCG hour3 / 10-hCG hour2 | *1.76 | 1.41 | 2.20 |
| 10-hCG hour1 / 10-hCG hour6 | *3.16 | 2.63 | 3.79 | 10-hCG hour3 / 5-hCG hour2 | *2.33 | 1.42 | 3.83 |
| 10-hCG hour1 / 5-hCG hour6 | 1.06 | 0.64 | 1.74 | 5-hCG hour3 / 10-hCG hour1 | 0.81 | 0.50 | 1.32 |
| 5-hCG hour1 / 10-hCG hour2 | 0.59 | 0.36 | 0.95 | 5-hCG hour3 / 5-hCG hour1 | *1.56 | 1.31 | 1.87 |
| 5-hCG hour1 / 5-hCG hour2 | 0.78 | 0.66 | 0.92 | 5-hCG hour3 / 10-hCG hour2 | 0.92 | 0.56 | 1.49 |
| 5-hCG hour1 / 10-hCG hour3 | 0.33 | 0.20 | 0.55 | 5-hCG hour3 / 5-hCG hour2 | *1.22 | 1.02 | 1.46 |
| 5-hCG hour1 / 5-hCG hour3 | 0.64 | 0.54 | 0.76 | 5-hCG hour3 / 10-hCG hour3 | 0.52 | 0.32 | 0.86 |
| 5-hCG hour1 / 10-hCG hour4 | 0.56 | 0.35 | 0.91 | 10-hCG hour4 / 10-hCG hour1 | 0.92 | 0.76 | 1.13 |
| 5-hCG hour1 / 5-hCG hour4 | 0.43 | 0.36 | 0.53 | 10-hCG hour4 / 5-hCG hour1 | *1.79 | 1.10 | 2.89 |
| 5-hCG hour1 / 10-hCG hour5 | 0.36 | 0.22 | 0.59 | 10-hCG hour4 / 10-hCG hour2 | 1.05 | 0.86 | 1.28 |
| 5-hCG hour1 / 5-hCG hour5 | 0.67 | 0.56 | 0.81 | 10-hCG hour4 / 5-hCG hour2 | 1.39 | 0.86 | 2.26 |
| 5-hCG hour1 / 10-hCG hour6 | *1.63 | 1.02 | 2.62 | 10-hCG hour4 / 10-hCG hour3 | 0.60 | 0.48 | 0.75 |
| 5-hCG hour1 / 5-hCG hour6 | 0.55 | 0.45 | 0.67 | 10-hCG hour4 / 5-hCG hour3 | 1.14 | 0.70 | 1.86 |
| 10-hCG hour2 / 5-hCG hour2 | 1.33 | 0.82 | 2.15 | 5-hCG hour4 / 10-hCG hour1 | 1.19 | 0.73 | 1.96 |
| 10-hCG hour2 / 10-hCG hour3 | 0.57 | 0.45 | 0.71 | 5-hCG hour4 / 5-hCG hour1 | *2.31 | 1.90 | 2.81 |
| 10-hCG hour2 / 5-hCG hour3 | 1.09 | 0.67 | 1.77 | 5-hCG hour4 / 10-hCG hour2 | 1.36 | 0.83 | 2.22 |
| 10-hCG hour2 / 10-hCG hour4 | 0.95 | 0.78 | 1.16 | 5-hCG hour4 / 5-hCG hour2 | *1.80 | 1.47 | 2.20 |
| 10-hCG hour2 / 5-hCG hour4 | 0.74 | 0.45 | 1.21 | 5-hCG hour4 / 10-hCG hour3 | 0.77 | 0.46 | 1.28 |
| 10-hCG hour2 / 10-hCG hour5 | 0.62 | 0.49 | 0.77 | 5-hCG hour4 / 5-hCG hour3 | *1.48 | 1.20 | 1.81 |
| 10-hCG hour2 / 5-hCG hour5 | 1.14 | 0.70 | 1.86 | 5-hCG hour4 / 10-hCG hour4 | 1.29 | 0.79 | 2.11 |
| 10-hCG hour2 / 10-hCG hour6 | *2.78 | 2.33 | 3.32 | 10-hCG hour5 / 10-hCG hour1 | *1.43 | 1.14 | 1.78 |
| 10-hCG hour2 / 5-hCG hour6 | 0.93 | 0.57 | 1.53 | 10-hCG hour5 / 5-hCG hour1 | *2.76 | 1.69 | 4.52 |
| 5-hCG hour2 / 10-hCG hour3 | 0.43 | 0.26 | 0.70 | 10-hCG hour5 / 10-hCG hour2 | *1.62 | 1.30 | 2.02 |
| 5-hCG hour2 / 5-hCG hour3 | 0.82 | 0.68 | 0.98 | 10-hCG hour5 / 5-hCG hour2 | *2.15 | 1.31 | 3.53 |
| 5-hCG hour2 / 10-hCG hour4 | 0.72 | 0.44 | 1.16 | 10-hCG hour5 / 10-hCG hour3 | 0.92 | 0.72 | 1.18 |
| 5-hCG hour2 / 5-hCG hour4 | 0.56 | 0.45 | 0.68 | 10-hCG hour5 / 5-hCG hour3 | *1.77 | 1.07 | 2.90 |
| 5-hCG hour2 / 10-hCG hour5 | 0.46 | 0.28 | 0.76 | 10-hCG hour5 / 10-hCG hour4 | *1.54 | 1.23 | 1.93 |
| 5-hCG hour2 / 5-hCG hour5 | 0.86 | 0.71 | 1.04 | 10-hCG hour5 / 5-hCG hour4 | 1.20 | 0.72 | 1.98 |
| 5-hCG hour2 / 10-hCG hour6 | *2.09 | 1.30 | 3.37 | 5-hCG hour5 / 10-hCG hour1 | 0.77 | 0.47 | 1.26 |
| 5-hCG hour2 / 5-hCG hour6 | 0.70 | 0.57 | 0.86 | 5-hCG hour5 / 5-hCG hour1 | *1.49 | 1.24 | 1.79 |
| 10-hCG hour3 / 5-hCG hour3 | 1.91 | 1.16 | 3.15 | 5-hCG hour5 / 10-hCG hour2 | 0.88 | 0.54 | 1.43 |
| 10-hCG hour3 / 10-hCG hour4 | *1.67 | 1.34 | 2.10 | 5-hCG hour5 / 5-hCG hour2 | 1.16 | 0.96 | 1.40 |
| 10-hCG hour3 / 5-hCG hour4 | 1.30 | 0.78 | 2.15 | 5-hCG hour5 / 10-hCG hour3 | 0.50 | 0.30 | 0.82 |
| 10-hCG hour3 / 10-hCG hour5 | 1.08 | 0.85 | 1.39 | 5-hCG hour5 / 5-hCG hour3 | 0.95 | 0.78 | 1.16 |
| 10-hCG hour3 / 5-hCG hour5 | *2.01 | 1.22 | 3.31 | 5-hCG hour5 / 10-hCG hour4 | 0.83 | 0.51 | 1.36 |
| 10-hCG hour3 / 10-hCG hour6 | *4.88 | 3.97 | 6.01 | 5-hCG hour5 / 5-hCG hour4 | 0.65 | 0.52 | 0.80 |
| 10-hCG hour3 / 5-hCG hour6 | 1.64 | 0.99 | 2.72 | 5-hCG hour5 / 10-hCG hour5 | 0.54 | 0.33 | 0.89 |
| 5-hCG hour3 / 10-hCG hour4 | 0.87 | 0.54 | 1.42 | 10-hCG hour6 / 10-hCG hour1 | 0.32 | 0.26 | 0.38 |
| 5-hCG hour3 / 5-hCG hour4 | 0.68 | 0.55 | 0.83 | 10-hCG hour6 / 5-hCG hour1 | 0.61 | 0.38 | 0.98 |
| 5-hCG hour3 / 10-hCG hour5 | 0.57 | 0.34 | 0.93 | 10-hCG hour6 / 10-hCG hour2 | 0.36 | 0.30 | 0.43 |
| 5-hCG hour3 / 5-hCG hour5 | 1.05 | 0.86 | 1.28 | 10-hCG hour6 / 5-hCG hour2 | 0.48 | 0.30 | 0.77 |
| 5-hCG hour3 / 10-hCG hour6 | *2.55 | 1.58 | 4.12 | 10-hCG hour6 / 10-hCG hour3 | 0.20 | 0.17 | 0.25 |
| 5-hCG hour3 / 5-hCG hour6 | 0.86 | 0.69 | 1.05 | 10-hCG hour6 / 5-hCG hour3 | 0.39 | 0.24 | 0.63 |
| 10-hCG hour4 / 5-hCG hour4 | 0.78 | 0.47 | 1.27 | 10-hCG hour6 / 10-hCG hour4 | 0.34 | 0.29 | 0.41 |
| 10-hCG hour4 / 10-hCG hour5 | 0.65 | 0.52 | 0.81 | 10-hCG hour6 / 5-hCG hour4 | 0.27 | 0.16 | 0.43 |
| 10-hCG hour4 / 5-hCG hour5 | 1.20 | 0.74 | 1.96 | 10-hCG hour6 / 10-hCG hour5 | 0.22 | 0.18 | 0.27 |
| 10-hCG hour4 / 10-hCG hour6 | *2.92 | 2.44 | 3.49 | 10-hCG hour6 / 5-hCG hour5 | 0.41 | 0.25 | 0.66 |
| 10-hCG hour4 / 5-hCG hour6 | 0.98 | 0.60 | 1.60 | 5-hCG hour6 / 10-hCG hour1 | 0.94 | 0.58 | 1.55 |
| 5-hCG hour4 / 10-hCG hour5 | 0.84 | 0.50 | 1.38 | 5-hCG hour6 / 5-hCG hour1 | *1.83 | 1.50 | 2.23 |
| 5-hCG hour4 / 5-hCG hour5 | *1.55 | 1.25 | 1.91 | 5-hCG hour6 / 10-hCG hour2 | 1.07 | 0.65 | 1.76 |
| 5-hCG hour4 / 10-hCG hour6 | *3.76 | 2.32 | 6.12 | 5-hCG hour6 / 5-hCG hour2 | *1.43 | 1.16 | 1.75 |
| 5-hCG hour4 / 5-hCG hour6 | *1.26 | 1.01 | 1.58 | 5-hCG hour6 / 10-hCG hour3 | 0.61 | 0.37 | 1.01 |
| 10-hCG hour5 / 5-hCG hour5 | *1.85 | 1.12 | 3.05 | 5-hCG hour6 / 5-hCG hour3 | 1.17 | 0.95 | 1.44 |
| 10-hCG hour5 / 10-hCG hour6 | *4.50 | 3.67 | 5.53 | 5-hCG hour6 / 10-hCG hour4 | 1.02 | 0.62 | 1.68 |
| 10-hCG hour5 / 5-hCG hour6 | 1.51 | 0.91 | 2.50 | 5-hCG hour6 / 5-hCG hour4 | 0.79 | 0.63 | 0.99 |
| 5-hCG hour5 / 10-hCG hour6 | *2.43 | 1.51 | 3.93 | 5-hCG hour6 / 10-hCG hour5 | 0.66 | 0.40 | 1.10 |
| 5-hCG hour5 / 5-hCG hour6 | 0.82 | 0.66 | 1.01 | 5-hCG hour6 / 5-hCG hour5 | 1.23 | 0.99 | 1.52 |
| 10-hCG hour6 / 5-hCG hour6 | 0.34 | 0.21 | 0.54 | 5-hCG hour6 / 10-hCG hour6 | *2.98 | 1.84 | 4.85 |

^1^Lower confidence limit

^2^Upper confidence limit

- Significant pairwise comparisons

**Supplementary Table 3*:*** *Pairwise Odds Ratios (ORs) and 95% Confidence Intervals for Total Sperm Motility in Litoria littlejohni, Comparing Different hCG Doses and Time Since Hormone Administration (n = 10 per treatment).*

| **Total Sperm Motility** | | | | | | | |
| --- | --- | --- | --- | --- | --- | --- | --- |
| **Contrast** | **Ratio** | **LCL^1^** | **UCL^2^** | **Reverse Contrast** | **Ratio** | **LCL^1^** | **UCL^2^** |
| 10-hCG hour1/5-hCG hour1 | 1.48 | 0.86 | 2.56 | 5-hCGhour1/10-hCGhour1 | 0.68 | 0.39 | 1.17 |
| 10-hCG hour1/10-hCG hour2 | *1.32 | 1.09 | 1.59 | 10-hCGhour2/10-hCGhour1 | 0.76 | 0.63 | 0.92 |
| 10-hCG hour1/5-hCG hour2 | 0.94 | 0.54 | 1.63 | 10-hCGhour2/5-hCGhour1 | 1.12 | 0.65 | 1.93 |
| 10-hCG hour1/10-hCG hour3 | 0.91 | 0.74 | 1.11 | 5-hCGhour2/10-hCGhour1 | 1.07 | 0.61 | 1.85 |
| 10-hCG hour1/5-hCG hour3 | 1.13 | 0.66 | 1.96 | 5-hCGhour2/5-hCGhour1 | *1.58 | 1.32 | 1.89 |
| 10-hCG hour1/10-hCG hour4 | 1.07 | 0.88 | 1.29 | 5-hCGhour2/10-hCGhour2 | 1.40 | 0.81 | 2.43 |
| 10-hCG hour1/5-hCG hour4 | 1.28 | 0.74 | 2.22 | 10-hCGhour3/10-hCGhour1 | 1.10 | 0.90 | 1.34 |
| 10-hCG hour1/10-hCG hour5 | *1.45 | 1.20 | 1.75 | 10-hCGhour3/5-hCGhour1 | 1.63 | 0.94 | 2.81 |
| 10-hCG hour1/5-hCG hour5 | *2.12 | 1.23 | 3.66 | 10-hCGhour3/10-hCGhour2 | *1.45 | 1.19 | 1.75 |
| 10-hCG hour1/10-hCG hour6 | *1.73 | 1.44 | 2.08 | 10-hCGhour3/5-hCGhour2 | 1.03 | 0.59 | 1.79 |
| 10-hCG hour1/5-hCG hour6 | *2.98 | 1.73 | 5.13 | 5-hCGhour3/10-hCGhour1 | 0.88 | 0.51 | 1.52 |
| 5-hCG hour1/10-hCG hour2 | 0.89 | 0.52 | 1.53 | 5-hCGhour3/5-hCGhour1 | *1.30 | 1.10 | 1.55 |
| 5-hCG hour1/5-hCG hour2 | 0.63 | 0.53 | 0.76 | 5-hCGhour3/10-hCGhour2 | 1.16 | 0.67 | 2.00 |
| 5-hCG hour1/10-hCG hour3 | 0.61 | 0.36 | 1.06 | 5-hCGhour3/5-hCGhour2 | 0.83 | 0.68 | 1.00 |
| 5-hCG hour1/5-hCG hour3 | 0.77 | 0.65 | 0.91 | 5-hCGhour3/10-hCGhour3 | 0.80 | 0.46 | 1.39 |
| 5-hCG hour1/10-hCG hour4 | 0.72 | 0.42 | 1.24 | 10-hCGhour4/10-hCGhour1 | 0.94 | 0.77 | 1.14 |
| 5-hCG hour1/5-hCG hour4 | 0.87 | 0.73 | 1.03 | 10-hCGhour4/5-hCGhour1 | 1.39 | 0.81 | 2.39 |
| 5-hCG hour1/10-hCG hour5 | 0.98 | 0.57 | 1.68 | 10-hCGhour4/10-hCGhour2 | 1.23 | 1.03 | 1.48 |
| 5-hCG hour1/5-hCG hour5 | *1.43 | 1.21 | 1.69 | 10-hCGhour4/5-hCGhour2 | 0.88 | 0.51 | 1.52 |
| 5-hCG hour1/10-hCG hour6 | 1.17 | 0.68 | 2.01 | 10-hCGhour4/10-hCGhour3 | 0.85 | 0.70 | 1.03 |
| 5-hCG hour1/5-hCG hour6 | *2.01 | 1.71 | 2.36 | 10-hCGhour4/5-hCGhour3 | 1.06 | 0.62 | 1.83 |
| 10-hCG hour2/5-hCG hour2 | 0.71 | 0.41 | 1.23 | 5-hCGhour4/10-hCGhour1 | 0.78 | 0.45 | 1.35 |
| 10-hCG hour2/10-hCG hour3 | 0.69 | 0.57 | 0.84 | 5-hCGhour4/5-hCGhour1 | 1.15 | 0.97 | 1.37 |
| 10-hCG hour2/5-hCG hour3 | 0.86 | 0.50 | 1.49 | 5-hCGhour4/10-hCGhour2 | 1.03 | 0.59 | 1.77 |
| 10-hCG hour2/10-hCG hour4 | 0.81 | 0.68 | 0.97 | 5-hCGhour4/5-hCGhour2 | 0.73 | 0.60 | 0.88 |
| 10-hCG hour2/5-hCG hour4 | 0.97 | 0.56 | 1.68 | 5-hCGhour4/10-hCGhour3 | 0.71 | 0.41 | 1.23 |
| 10-hCG hour2/10-hCG hour5 | 1.10 | 0.92 | 1.32 | 5-hCGhour4/5-hCGhour3 | 0.89 | 0.74 | 1.06 |
| 10-hCG hour2/5-hCG hour5 | 1.61 | 0.94 | 2.77 | 5-hCGhour4/10-hCGhour4 | 0.83 | 0.48 | 1.44 |
| 10-hCG hour2/10-hCG hour6 | *1.31 | 1.10 | 1.57 | 10-hCGhour5/10-hCGhour1 | 0.69 | 0.57 | 0.83 |
| 10-hCG hour2/5-hCG hour6 | *2.26 | 1.32 | 3.88 | 10-hCGhour5/5-hCGhour1 | 1.02 | 0.59 | 1.76 |
| 5-hCG hour2/10-hCG hour3 | 0.97 | 0.56 | 1.69 | 10-hCGhour5/10-hCGhour2 | 0.91 | 0.76 | 1.09 |
| 5-hCG hour2/5-hCG hour3 | *1.21 | 1.00 | 1.46 | 10-hCGhour5/5-hCGhour2 | 0.65 | 0.37 | 1.12 |
| 5-hCG hour2/10-hCG hour4 | 1.14 | 0.66 | 1.97 | 10-hCGhour5/10-hCGhour3 | 0.63 | 0.52 | 0.76 |
| 5-hCG hour2/5-hCG hour4 | *1.37 | 1.13 | 1.65 | 10-hCGhour5/5-hCGhour3 | 0.78 | 0.45 | 1.35 |
| 5-hCG hour2/10-hCG hour5 | 1.55 | 0.89 | 2.67 | 10-hCGhour5/10-hCGhour4 | 0.74 | 0.62 | 0.88 |
| 5-hCG hour2/5-hCG hour5 | *2.26 | 1.89 | 2.71 | 10-hCGhour5/5-hCGhour4 | 0.89 | 0.51 | 1.53 |
| 5-hCG hour2/10-hCG hour6 | *1.85 | 1.07 | 3.19 | 5-hCGhour5/10-hCGhour1 | 0.47 | 0.27 | 0.82 |
| 5-hCG hour2/5-hCG hour6 | *3.18 | 2.66 | 3.79 | 5-hCGhour5/5-hCGhour1 | 0.70 | 0.59 | 0.82 |
| 10-hCG hour3/5-hCG hour3 | 1.25 | 0.72 | 2.16 | 5-hCGhour5/10-hCGhour2 | 0.62 | 0.36 | 1.07 |
| 10-hCG hour3/10-hCG hour4 | 1.17 | 0.97 | 1.42 | 5-hCGhour5/5-hCGhour2 | 0.44 | 0.37 | 0.53 |
| 10-hCG hour3/5-hCGhour4 | 1.41 | 0.81 | 2.44 | 5-hCGhour5/10-hCGhour3 | 0.43 | 0.25 | 0.74 |
| 10-hCG hour3/10-hCGhour5 | *1.59 | 1.32 | 1.92 | 5-hCGhour5/5-hCGhour3 | 0.54 | 0.45 | 0.64 |
| 10-hCG hour3/5-hCGhour5 | *2.33 | 1.35 | 4.02 | 5-hCGhour5/10-hCGhour4 | 0.50 | 0.29 | 0.87 |
| 10-hCG hour3/10-hCG hour6 | *1.90 | 1.58 | 2.29 | 5-hCGhour5/5-hCGhour4 | 0.61 | 0.51 | 0.72 |
| 10-hCG hour3/5-hCG hour6 | *3.27 | 1.90 | 5.64 | 5-hCGhour5/10-hCGhour5 | 0.68 | 0.40 | 1.18 |
| 5-hCG hour3/10-hCG hour4 | 0.94 | 0.55 | 1.62 | 10-hCGhour6/10-hCGhour1 | 0.58 | 0.48 | 0.70 |
| 5-hCG hour3/5-hCG hour4 | 1.13 | 0.94 | 1.35 | 10-hCGhour6/5-hCGhour1 | 0.86 | 0.50 | 1.47 |
| 5-hCG hour3/10-hCG hour5 | 1.28 | 0.74 | 2.20 | 10-hCGhour6/10-hCGhour2 | 0.76 | 0.64 | 0.91 |
| 5-hCG hour3/5-hCG hour5 | *1.87 | 1.57 | 2.22 | 10-hCGhour6/5-hCGhour2 | 0.54 | 0.31 | 0.93 |
| 5-hCG hour3/10-hCG hour6 | 1.52 | 0.89 | 2.62 | 10-hCGhour6/10-hCGhour3 | 0.53 | 0.44 | 0.63 |
| 5-hCG hour3/5-hCG hour6 | *2.62 | 2.22 | 3.10 | 10-hCGhour6/5-hCGhour3 | 0.66 | 0.38 | 1.13 |
| 10-hCG hour4/5-hCG hour4 | 1.20 | 0.70 | 2.07 | 10-hCGhour6/10-hCGhour4 | 0.62 | 0.52 | 0.74 |
| 10-hCG hour4/10-hCG hour5 | *1.36 | 1.13 | 1.62 | 10-hCGhour6/5-hCGhour4 | 0.74 | 0.43 | 1.28 |
| 10-hCG hour4/5-hCG hour5 | 1.98 | 1.15 | 3.41 | 10-hCGhour6/10-hCGhour5 | 0.84 | 0.70 | 0.99 |
| 10-hCG hour4/10-hCG hour6 | *1.62 | 1.36 | 1.93 | 10-hCGhour6/5-hCGhour5 | 1.22 | 0.71 | 2.10 |
| 10-hCG hour4/5-hCG hour6 | *2.79 | 1.62 | 4.79 | 5-hCGhour6/10-hCGhour1 | 0.34 | 0.19 | 0.58 |
| 5-hCG hour4/10-hCG hour5 | 1.13 | 0.66 | 1.95 | 5-hCGhour6/5-hCGhour1 | 0.50 | 0.42 | 0.58 |
| 5-hCG hour4/5-hCG hour5 | *1.65 | 1.39 | 1.96 | 5-hCGhour6/10-hCGhour2 | 0.44 | 0.26 | 0.76 |
| 5-hCG hour4/10-hCG hour6 | 1.35 | 0.78 | 2.32 | 5-hCGhour6/5-hCGhour2 | 0.31 | 0.26 | 0.38 |
| 5-hCG hour4/5-hCG hour6 | *2.32 | 1.96 | 2.74 | 5-hCGhour6/10-hCGhour3 | 0.31 | 0.18 | 0.53 |
| 10-hCG hour5/5-hCG hour5 | 1.46 | 0.85 | 2.51 | 5-hCGhour6/5-hCGhour3 | 0.38 | 0.32 | 0.45 |
| 10-hCG hour5/10-hCG hour6 | *1.19 | 1.01 | 1.42 | 5-hCGhour6/10-hCGhour4 | 0.36 | 0.21 | 0.62 |
| 10-hCG hour5/5-hCG hour6 | *2.06 | 1.20 | 3.53 | 5-hCGhour6/5-hCGhour4 | 0.43 | 0.36 | 0.51 |
| 5-hCG hour5/10-hCG hour6 | 0.82 | 0.48 | 1.40 | 5-hCGhour6/10-hCGhour5 | 0.49 | 0.28 | 0.83 |
| 5-hCG hour5/5-hCG hour6 | *1.41 | 1.20 | 1.64 | 5-hCGhour6/5-hCGhour5 | 0.71 | 0.61 | 0.83 |
| 10-hCG hour6/5-hCG hour6 | *1.72 | 1.00 | 2.95 | 5-hCGhour6/10-hCGhour6 | 0.58 | 0.34 | 1.00 |

^1^Lower confidence limit

^2^Upper confidence limit * Significant pairwise comparisons

**Supplementary Table 4:** *Pairwise Odds Ratios (ORs) and 95% Confidence Intervals for Forward Progressive Sperm Motility in Litoria littlejohni, Comparing Different hCG Doses and Time Since Hormone Administration (n = 10 per treatment).*

| **Forward Progressive Sperm Motility** | | | | | | | | |
| --- | --- | --- | --- | --- | --- | --- | --- | --- |
| **Contrast** | **Ratio** | **LCL^1^** | **UCL^2^** | **Reverse Contrast** | **Ratio** | **LCL^1^** | **UCL^2^** |  |
| 10-hCGhour1/5-hCGhour1 | 0.77 | 0.38 | 1.56 | 5-hCGhour1/10-hCGhour1 | 1.30 | 0.64 | 2.62 |  |
| 10-hCGhour1/10-hCGhour2 | *1.23 | 1.06 | 1.42 | 10-hCGhour2/10-hCGhour1 | 0.81 | 0.71 | 0.94 |  |
| 10-hCGhour1/5-hCGhour2 | 0.65 | 0.32 | 1.31 | 10-hCGhour2/5-hCGhour1 | 0.63 | 0.31 | 1.27 |  |
| 10-hCGhour1/10-hCGhour3 | *1.17 | 1.01 | 1.34 | 5-hCGhour2/10-hCGhour1 | 1.54 | 0.76 | 3.11 |  |
| 10-hCGhour1/5-hCGhour3 | 0.89 | 0.44 | 1.79 | 5-hCGhour2/5-hCGhour1 | *1.19 | 1.05 | 1.35 |  |
| 10-hCGhour1/10-hCGhour4 | *1.48 | 1.28 | 1.71 | 5-hCGhour2/10-hCGhour2 | 1.89 | 0.93 | 3.81 |  |
| 10-hCGhour1/5-hCGhour4 | 0.82 | 0.41 | 1.65 | 10-hCGhour3/10-hCGhour1 | 0.86 | 0.74 | 0.99 |  |
| 10-hCGhour1/10-hCGhour5 | *1.38 | 1.20 | 1.59 | 10-hCGhour3/5-hCGhour1 | 0.66 | 0.33 | 1.34 |  |
| 10-hCGhour1/5-hCGhour5 | 0.97 | 0.48 | 1.96 | 10-hCGhour3/10-hCGhour2 | 1.05 | 0.91 | 1.21 |  |
| 10-hCGhour1/10-hCGhour6 | *2.31 | 1.99 | 2.67 | 10-hCGhour3/5-hCGhour2 | 0.56 | 0.28 | 1.13 |  |
| 10-hCGhour1/5-hCGhour6 | 1.35 | 0.67 | 2.74 | 5-hCGhour3/10-hCGhour1 | 1.13 | 0.56 | 2.28 |  |
| 5-hCGhour1/10-hCGhour2 | 1.59 | 0.79 | 3.21 | 5-hCGhour3/5-hCGhour1 | 0.87 | 0.77 | 0.99 |  |
| 5-hCGhour1/5-hCGhour2 | 0.84 | 0.74 | 0.96 | 5-hCGhour3/10-hCGhour2 | 1.38 | 0.69 | 2.79 |  |
| 5-hCGhour1/10-hCGhour3 | 1.51 | 0.75 | 3.05 | 5-hCGhour3/5-hCGhour2 | 0.73 | 0.65 | 0.83 |  |
| 5-hCGhour1/5-hCGhour3 | *1.15 | 1.01 | 1.30 | 5-hCGhour3/10-hCGhour3 | 1.31 | 0.65 | 2.65 |  |
| 5-hCGhour1/10-hCGhour4 | 1.92 | 0.95 | 3.87 | 10-hCGhour4/10-hCGhour1 | 0.67 | 0.59 | 0.78 |  |
| 5-hCGhour1/5-hCGhour4 | 1.06 | 0.93 | 1.21 | 10-hCGhour4/5-hCGhour1 | 0.52 | 0.26 | 1.05 |  |
| 5-hCGhour1/10-hCGhour5 | 1.79 | 0.89 | 3.61 | 10-hCGhour4/10-hCGhour2 | 0.83 | 0.72 | 0.96 |  |
| 5-hCGhour1/5-hCGhour5 | *1.26 | 1.10 | 1.43 | 10-hCGhour4/5-hCGhour2 | 0.44 | 0.22 | 0.89 |  |
| 5-hCGhour1/10-hCGhour6 | *2.99 | 1.48 | 6.04 | 10-hCGhour4/10-hCGhour3 | 0.79 | 0.68 | 0.91 |  |
| 5-hCGhour1/5-hCGhour6 | *1.75 | 1.54 | 2.00 | 10-hCGhour4/5-hCGhour3 | 0.60 | 0.30 | 1.21 |  |
| 10-hCGhour2/5-hCGhour2 | 0.53 | 0.26 | 1.07 | 5-hCGhour4/10-hCGhour1 | 1.22 | 0.60 | 2.47 |  |
| 10-hCGhour2/10-hCGhour3 | 0.95 | 0.82 | 1.10 | 5-hCGhour4/5-hCGhour1 | 0.94 | 0.83 | 1.07 |  |
| 10-hCGhour2/5-hCGhour3 | 0.72 | 0.36 | 1.46 | 5-hCGhour4/10-hCGhour2 | 1.50 | 0.74 | 3.03 |  |
| 10-hCGhour2/10-hCGhour4 | *1.21 | 1.05 | 1.39 | 5-hCGhour4/5-hCGhour2 | 0.79 | 0.70 | 0.90 |  |
| 10-hCGhour2/5-hCGhour4 | 0.67 | 0.33 | 1.35 | 5-hCGhour4/10-hCGhour3 | 1.42 | 0.70 | 2.88 |  |
| 10-hCGhour2/10-hCGhour5 | 1.13 | 0.98 | 1.30 | 5-hCGhour4/5-hCGhour3 | 1.08 | 0.95 | 1.23 |  |
| 10-hCGhour2/5-hCGhour5 | 0.79 | 0.39 | 1.60 | 5-hCGhour4/10-hCGhour4 | 1.81 | 0.90 | 3.66 |  |
| 10-hCGhour2/10-hCGhour6 | 1.88 | 1.62 | 2.17 | 10-hCGhour5/10-hCGhour1 | 0.72 | 0.63 | 0.84 |  |
| 10-hCGhour2/5-hCGhour6 | 1.10 | 0.55 | 2.23 | 10-hCGhour5/5-hCGhour1 | 0.56 | 0.28 | 1.13 |  |
| 5-hCGhour2/10-hCGhour3 | 1.79 | 0.89 | 3.62 | 10-hCGhour5/10-hCGhour2 | 0.89 | 0.77 | 1.03 |  |
| 5-hCGhour2/5-hCGhour3 | *1.36 | 1.20 | 1.55 | 10-hCGhour5/5-hCGhour2 | 0.47 | 0.23 | 0.95 |  |
| 5-hCGhour2/10-hCGhour4 | *2.28 | 1.13 | 4.60 | 10-hCGhour5/10-hCGhour3 | 0.84 | 0.73 | 0.97 |  |
| 5-hCGhour2/5-hCGhour4 | *1.26 | 1.11 | 1.43 | 10-hCGhour5/5-hCGhour3 | 0.64 | 0.32 | 1.30 |  |
| 5-hCGhour2/10-hCGhour5 | *2.13 | 1.05 | 4.29 | 10-hCGhour5/10-hCGhour4 | 1.07 | 0.93 | 1.24 |  |
| 5-hCGhour2/5-hCGhour5 | *1.49 | 1.31 | 1.70 | 10-hCGhour5/5-hCGhour4 | 0.59 | 0.29 | 1.20 |  |
| 5-hCGhour2/10-hCGhour6 | *3.55 | 1.75 | 7.17 | 5-hCGhour5/10-hCGhour1 | 1.03 | 0.51 | 2.08 |  |
| 5-hCGhour2/5-hCGhour6 | *2.08 | 1.82 | 2.38 | 5-hCGhour5/5-hCGhour1 | 0.80 | 0.70 | 0.91 |  |
| 10-hCGhour3/5-hCGhour3 | 0.76 | 0.38 | 1.54 | 5-hCGhour5/10-hCGhour2 | 1.26 | 0.63 | 2.56 |  |
| 10-hCGhour3/10-hCGhour4 | *1.27 | 1.10 | 1.47 | 5-hCGhour5/5-hCGhour2 | 0.67 | 0.59 | 0.76 |  |
| 10-hCGhour3/5-hCGhour4 | 0.70 | 0.35 | 1.42 | 5-hCGhour5/10-hCGhour3 | 1.20 | 0.59 | 2.43 |  |
| 10-hCGhour3/10-hCGhour5 | *1.19 | 1.03 | 1.37 | 5-hCGhour5/5-hCGhour3 | 0.91 | 0.80 | 1.04 |  |
| 10-hCGhour3/5-hCGhour5 | 0.83 | 0.41 | 1.68 | 5-hCGhour5/10-hCGhour4 | 1.53 | 0.76 | 3.09 |  |
| 10-hCGhour3/10-hCGhour6 | *1.98 | 1.71 | 2.29 | 5-hCGhour5/5-hCGhour4 | 0.84 | 0.74 | 0.96 |  |
| 10-hCGhour3/5-hCGhour6 | 1.16 | 0.57 | 2.35 | 5-hCGhour5/10-hCGhour5 | 1.42 | 0.70 | 2.88 |  |
| 5-hCGhour3/10-hCGhour4 | 1.67 | 0.83 | 3.37 | 10-hCGhour6/10-hCGhour1 | 0.43 | 0.37 | 0.50 |  |
| 5-hCGhour3/5-hCGhour4 | 0.92 | 0.81 | 1.05 | 10-hCGhour6/5-hCGhour1 | 0.33 | 0.17 | 0.68 |  |
| 5-hCGhour3/10-hCGhour5 | 1.56 | 0.77 | 3.14 | 10-hCGhour6/10-hCGhour2 | 0.53 | 0.46 | 0.62 |  |
| 5-hCGhour3/5-hCGhour5 | 1.09 | 0.96 | 1.25 | 10-hCGhour6/5-hCGhour2 | 0.28 | 0.14 | 0.57 |  |
| 5-hCGhour3/10-hCGhour6 | *2.60 | 1.29 | 5.25 | 10-hCGhour6/10-hCGhour3 | 0.51 | 0.44 | 0.59 |  |
| 5-hCGhour3/5-hCGhour6 | *1.53 | 1.34 | 1.74 | 10-hCGhour6/5-hCGhour3 | 0.38 | 0.19 | 0.78 |  |
| 10-hCGhour4/5-hCGhour4 | 0.55 | 0.27 | 1.12 | 10-hCGhour6/10-hCGhour4 | 0.64 | 0.56 | 0.74 |  |
| 10-hCGhour4/10-hCGhour5 | 0.93 | 0.81 | 1.08 | 10-hCGhour6/5-hCGhour4 | 0.36 | 0.18 | 0.72 |  |
| 10-hCGhour4/5-hCGhour5 | 0.66 | 0.32 | 1.32 | 10-hCGhour6/10-hCGhour5 | 0.60 | 0.52 | 0.69 |  |
| 10-hCGhour4/10-hCGhour6 | *1.56 | 1.35 | 1.80 | 10-hCGhour6/5-hCGhour5 | 0.42 | 0.21 | 0.85 |  |
| 10-hCGhour4/5-hCGhour6 | 0.91 | 0.45 | 1.85 | 5-hCGhour6/10-hCGhour1 | 0.74 | 0.37 | 1.49 |  |
| 5-hCGhour4/10-hCGhour5 | 1.69 | 0.83 | 3.41 | 5-hCGhour6/5-hCGhour1 | 0.57 | 0.50 | 0.65 |  |
| 5-hCGhour4/5-hCGhour5 | *1.19 | 1.04 | 1.35 | 5-hCGhour6/10-hCGhour2 | 0.91 | 0.45 | 1.83 |  |
| 5-hCGhour4/10-hCGhour6 | *2.82 | 1.39 | 5.70 | 5-hCGhour6/5-hCGhour2 | 0.48 | 0.42 | 0.55 |  |
| 5-hCGhour4/5-hCGhour6 | *1.65 | 1.45 | 1.89 | 5-hCGhour6/10-hCGhour3 | 0.86 | 0.43 | 1.74 |  |
| 10-hCGhour5/5-hCGhour5 | 0.70 | 0.35 | 1.42 | 5-hCGhour6/5-hCGhour3 | 0.66 | 0.57 | 0.75 |  |
| 10-hCGhour5/10-hCGhour6 | *1.67 | 1.44 | 1.93 | 5-hCGhour6/10-hCGhour4 | 1.09 | 0.54 | 2.21 |  |
| 10-hCGhour5/5-hCGhour6 | 0.98 | 0.48 | 1.98 | 5-hCGhour6/5-hCGhour4 | 0.60 | 0.53 | 0.69 |  |
| 5-hCGhour5/10-hCGhour6 | *2.38 | 1.17 | 4.81 | 5-hCGhour6/10-hCGhour5 | 1.02 | 0.50 | 2.06 |  |
| 5-hCGhour5/5-hCGhour6 | *1.39 | 1.22 | 1.60 | 5-hCGhour6/5-hCGhour5 | 0.72 | 0.63 | 0.82 |  |
| 10-hCGhour6/5-hCGhour6 | 0.59 | 0.29 | 1.19 | 5-hCGhour6/10-hCGhour6 | 1.70 | 0.84 | 3.45 |  |

^1^Lower confidence limit

^2^Upper confidence limit * Significant pairwise comparisons

**Supplementary Table 5:** *Pairwise Odds Ratios (ORs) and 95% Confidence Intervals for Sperm Concentration and Membrane Integrity in Litoria littlejohni, Comparing 12-Month Time Points (n = 10 per treatment).*

| **Sperm Concentration** | | | | | | | | **Membrane Integrity** | | | | | | | |
| --- | --- | --- | --- | --- | --- | --- | --- | --- | --- | --- | --- | --- | --- | --- | --- |
| **Contrast** | **Ratio** | **LCL^1^** | **UCL^2^** | **Reverse Contrast** | **Ratio** | **LCL^1^** | **UCL^2^** | **Contrast** | **Ratio** | **LCL^1^** | **UCL^2^** | **Reverse Contrast** | **Ratio** | **LCL^1^** | **UCL^2^** |
| Apr / Aug | 0.41 | 0.17 | 0.97 | Aug / Apr | *2.46 | 1.03 | 5.83 | Jan / Feb | 0.27 | 0.08 | 0.90 | Feb / Jan | *3.69 | 1.12 | 12.16 |
| Apr / Dec | 0.46 | 0.21 | 1.00 | Dec / Apr | *2.18 | 1.00 | 4.74 | Jan / Mar | 0.37 | 0.09 | 1.50 | Mar / Jan | *2.71 | 0.67 | 11.05 |
| Apr / Feb | 0.61 | 0.29 | 1.28 | Dec / Aug | 0.89 | 0.39 | 2.03 | Jan / Apr | 1.47 | 0.41 | 5.33 | Mar / Feb | 0.74 | 0.24 | 2.26 |
| Apr / Jan | 0.42 | 0.16 | 1.10 | Feb / Apr | 1.64 | 0.78 | 3.44 | Jan / May | 0.25 | 0.08 | 0.81 | Apr / Jan | 0.68 | 0.19 | 2.46 |
| Apr / Jul | 0.52 | 0.23 | 1.20 | Feb / Aug | 0.67 | 0.30 | 1.47 | Jan / Jun | 1.85 | 0.56 | 6.13 | Apr / Feb | 0.18 | 0.07 | 0.49 |
| Apr / Jun | 0.52 | 0.26 | 1.07 | Feb / Dec | 0.75 | 0.46 | 1.23 | Jan / Jul | 0.18 | 0.05 | 0.69 | Apr / Mar | 0.25 | 0.07 | 0.85 |
| Apr / Mar | 0.26 | 0.11 | 0.64 | Jan / Apr | 2.38 | 0.91 | 6.21 | Jan / Aug | 0.08 | 0.02 | 0.34 | May / Jan | *3.98 | 1.24 | 12.77 |
| Apr / May | 0.45 | 0.22 | 0.91 | Jan / Aug | 0.97 | 0.35 | 2.67 | Jan / Sep | 0.12 | 0.03 | 0.43 | May / Feb | 1.08 | 0.62 | 1.88 |
| Apr / Nov | 0.49 | 0.23 | 1.05 | Jan / Dec | 1.09 | 0.43 | 2.75 | Jan / Oct | 0.71 | 0.21 | 2.42 | May / Mar | 1.47 | 0.49 | 4.37 |
| Apr / Oct | 0.17 | 0.08 | 0.35 | Jan / Feb | 1.45 | 0.58 | 3.62 | Jan / Nov | 2.16 | 0.62 | 7.50 | May / Apr | *5.85 | 2.29 | 14.95 |
| Apr / Sep | 0.41 | 0.19 | 0.93 | Jul / Apr | 1.92 | 0.84 | 4.43 | Jan / Dec | 0.20 | 0.06 | 0.67 | Jun / Jan | 0.54 | 0.16 | 1.80 |
| Aug / Dec | 1.13 | 0.49 | 2.58 | Jul / Aug | 0.78 | 0.32 | 1.90 | Feb / Mar | 1.36 | 0.44 | 4.17 | Jun / Feb | 0.15 | 0.07 | 0.30 |
| Aug / Feb | 1.50 | 0.68 | 3.28 | Jul / Dec | 0.88 | 0.40 | 1.97 | Feb / Apr | *5.42 | 2.05 | 14.30 | Jun / Mar | 0.20 | 0.06 | 0.62 |
| Aug / Jan | 1.03 | 0.37 | 2.84 | Jul / Feb | 1.17 | 0.54 | 2.53 | Feb / May | 0.93 | 0.53 | 1.61 | Jun / Apr | 0.80 | 0.30 | 2.12 |
| Aug / Jul | 1.28 | 0.53 | 3.10 | Jul / Jan | 0.81 | 0.30 | 2.16 | Feb / Jun | *6.81 | 3.38 | 13.71 | Jun / May | 0.14 | 0.08 | 0.22 |
| Aug / Jun | 1.28 | 0.58 | 2.81 | Jun / Apr | 1.92 | 0.94 | 3.92 | Feb / Jul | 0.67 | 0.24 | 1.88 | Jul / Jan | *5.49 | 1.45 | 20.80 |
| Aug / Mar | 0.64 | 0.24 | 1.67 | Jun / Aug | 0.78 | 0.36 | 1.71 | Feb / Aug | 0.31 | 0.10 | 0.94 | Jul / Feb | 1.49 | 0.53 | 4.16 |
| Aug / May | 1.10 | 0.50 | 2.40 | Jun / Dec | 0.88 | 0.46 | 1.67 | Feb / Sep | 0.44 | 0.20 | 0.96 | Jul / Mar | 2.02 | 0.57 | 7.20 |
| Aug / Nov | 1.20 | 0.53 | 2.72 | Jun / Feb | 1.17 | 0.67 | 2.05 | Feb / Oct | *2.63 | 1.09 | 6.31 | Jul / Apr | *8.07 | 2.59 | 25.19 |
| Aug / Oct | 0.41 | 0.18 | 0.91 | Jun / Jan | 0.81 | 0.33 | 1.94 | Feb / Nov | *7.97 | 3.21 | 19.79 | Jul / May | 1.38 | 0.51 | 3.75 |
| Aug / Sep | 1.02 | 0.43 | 2.43 | Jun / Jul | 1.00 | 0.48 | 2.08 | Feb / Dec | 0.73 | 0.39 | 1.35 | Jul / Jun | *10.14 | 3.59 | 28.66 |
| Dec / Feb | 1.33 | 0.81 | 2.17 | Mar / Apr | 3.84 | 1.55 | 9.52 | Mar / Apr | *3.99 | 1.18 | 13.55 | Aug / Jan | *11.94 | 2.94 | 48.55 |
| Dec / Jan | 0.92 | 0.36 | 2.31 | Mar / Aug | 1.57 | 0.60 | 4.09 | Mar / May | 0.68 | 0.23 | 2.04 | Aug / Feb | *3.24 | 1.06 | 9.87 |
| Dec / Jul | 1.13 | 0.51 | 2.53 | Mar / Dec | 1.76 | 0.73 | 4.27 | Mar / Jun | *5.02 | 1.62 | 15.51 | Aug / Mar | *4.40 | 1.15 | 16.85 |
| Dec / Jun | 1.14 | 0.60 | 2.16 | Mar / Feb | 2.34 | 1.00 | 5.46 | Mar / Jul | 0.49 | 0.14 | 1.76 | Aug / Apr | *17.56 | 5.19 | 59.43 |
| Dec / Mar | 0.57 | 0.23 | 1.37 | Mar / Jan | 1.62 | 0.57 | 4.55 | Mar / Aug | 0.23 | 0.06 | 0.87 | Aug / May | *3.00 | 1.01 | 8.93 |
| Dec / May | 0.98 | 0.52 | 1.84 | Mar / Jul | 2.00 | 0.79 | 5.05 | Mar / Sep | 0.32 | 0.10 | 1.09 | Aug / Jun | *22.07 | 7.11 | 68.53 |
| Dec / Nov | 1.07 | 0.52 | 2.19 | Mar / Jun | 2.01 | 0.89 | 4.53 | Mar / Oct | 1.93 | 0.61 | 6.13 | Aug / Jul | 2.18 | 0.61 | 7.71 |
| Dec / Oct | 0.36 | 0.18 | 0.73 | May / Apr | 2.23 | 1.10 | 4.53 | Mar / Nov | *5.87 | 1.81 | 19.01 | Sep / Jan | *8.41 | 2.34 | 30.22 |
| Dec / Sep | 0.90 | 0.42 | 1.94 | May / Aug | 0.91 | 0.42 | 1.98 | Mar / Dec | 0.53 | 0.17 | 1.71 | Sep / Feb | *2.28 | 1.04 | 5.00 |
| Feb / Jan | 0.69 | 0.28 | 1.72 | May / Dec | 1.02 | 0.54 | 1.93 | Apr / May | 0.17 | 0.07 | 0.44 | Sep / Mar | 3.10 | 0.92 | 10.42 |
| Feb / Jul | 0.85 | 0.40 | 1.84 | May / Feb | 1.36 | 0.79 | 2.35 | Apr / Jun | 1.26 | 0.47 | 3.34 | Sep / Apr | *12.37 | 4.22 | 36.26 |
| Feb / Jun | 0.86 | 0.49 | 1.50 | May / Jan | 0.94 | 0.39 | 2.25 | Apr / Jul | 0.12 | 0.04 | 0.39 | Sep / May | *2.11 | 1.10 | 4.07 |
| Feb / Mar | 0.43 | 0.18 | 1.00 | May / Jul | 1.16 | 0.56 | 2.41 | Apr / Aug | 0.06 | 0.02 | 0.19 | Sep / Jun | *15.54 | 6.90 | 34.99 |
| Feb / May | 0.74 | 0.43 | 1.27 | May / Jun | 1.16 | 0.96 | 1.41 | Apr / Sep | 0.08 | 0.03 | 0.24 | Sep / Jul | 1.53 | 0.50 | 4.73 |
| Feb / Nov | 0.80 | 0.41 | 1.57 | May / Mar | 0.58 | 0.26 | 1.30 | Apr / Oct | 0.48 | 0.18 | 1.33 | Sep / Aug | 0.70 | 0.21 | 2.35 |
| Feb / Oct | 0.27 | 0.14 | 0.52 | Nov / Apr | 2.04 | 0.95 | 4.38 | Apr / Nov | 1.47 | 0.52 | 4.12 | Oct / Jan | 1.40 | 0.41 | 4.76 |
| Feb / Sep | 0.68 | 0.39 | 1.20 | Nov / Aug | 0.83 | 0.37 | 1.88 | Apr / Dec | 0.13 | 0.05 | 0.37 | Oct / Feb | 0.38 | 0.16 | 0.91 |
| Jan / Jul | 1.24 | 0.46 | 3.30 | Nov / Dec | 0.94 | 0.46 | 1.92 | May / Jun | *7.35 | 4.47 | 12.10 | Oct / Mar | 0.52 | 0.16 | 1.64 |
| Jan / Jun | 1.24 | 0.52 | 2.99 | Nov / Feb | 1.24 | 0.64 | 2.43 | May / Jul | 0.72 | 0.27 | 1.97 | Oct / Apr | 2.06 | 0.75 | 5.64 |
| Jan / Mar | 0.62 | 0.22 | 1.74 | Nov / Jan | 0.86 | 0.34 | 2.18 | May / Aug | 0.33 | 0.11 | 0.99 | Oct / May | 0.35 | 0.15 | 0.82 |
| Jan / May | 1.07 | 0.44 | 2.56 | Nov / Jul | 1.06 | 0.48 | 2.33 | May / Sep | 0.47 | 0.25 | 0.91 | Oct / Jun | *2.59 | 1.05 | 6.37 |
| Jan / Nov | 1.16 | 0.46 | 2.95 | Nov / Jun | 1.07 | 0.55 | 2.06 | May / Oct | *2.84 | 1.22 | 6.61 | Oct / Jul | 0.26 | 0.09 | 0.74 |
| Jan / Oct | 0.39 | 0.16 | 0.99 | Nov / Mar | 0.53 | 0.22 | 1.27 | May / Nov | *8.60 | 3.58 | 20.66 | Oct / Aug | 0.12 | 0.04 | 0.37 |
| Jan / Sep | 0.99 | 0.38 | 2.54 | Nov / May | 0.92 | 0.48 | 1.76 | May / Dec | 0.78 | 0.38 | 1.63 | Oct / Sep | 0.17 | 0.06 | 0.45 |
| Jul / Jun | 1.00 | 0.48 | 2.10 | Oct / Apr | *6.02 | 2.84 | 12.77 | Jun / Jul | 0.10 | 0.03 | 0.28 | Nov / Jan | 0.46 | 0.13 | 1.60 |
| Jul / Mar | 0.50 | 0.20 | 1.27 | Oct / Aug | *2.45 | 1.10 | 5.49 | Jun / Aug | 0.05 | 0.01 | 0.14 | Nov / Feb | 0.13 | 0.05 | 0.31 |
| Jul / May | 0.86 | 0.41 | 1.79 | Oct / Dec | *2.77 | 1.37 | 5.59 | Jun / Sep | 0.06 | 0.03 | 0.14 | Nov / Mar | 0.17 | 0.05 | 0.55 |
| Jul / Nov | 0.94 | 0.43 | 2.07 | Oct / Feb | *3.67 | 1.91 | 7.06 | Jun / Oct | 0.39 | 0.16 | 0.95 | Nov / Apr | 0.68 | 0.24 | 1.91 |
| Jul / Oct | 0.32 | 0.15 | 0.69 | Oct / Jan | *2.53 | 1.01 | 6.35 | Jun / Nov | 1.17 | 0.47 | 2.90 | Nov / May | 0.12 | 0.05 | 0.28 |
| Jul / Sep | 0.80 | 0.35 | 1.83 | Oct / Jul | *3.13 | 1.44 | 6.80 | Jun / Dec | 0.11 | 0.05 | 0.25 | Nov / Jun | 0.85 | 0.34 | 2.12 |
| Jun / Mar | 0.50 | 0.22 | 1.13 | Oct / Jun | *3.14 | 1.64 | 6.02 | Jul / Aug | 0.46 | 0.13 | 1.63 | Nov / Jul | 0.08 | 0.03 | 0.25 |
| Jun / May | 0.86 | 0.71 | 1.04 | Oct / Mar | 1.57 | 0.66 | 3.71 | Jul / Sep | 0.65 | 0.21 | 2.01 | Nov / Aug | 0.04 | 0.01 | 0.13 |
| Jun / Nov | 0.94 | 0.48 | 1.82 | Oct / May | *2.70 | 1.42 | 5.14 | Jul / Oct | *3.91 | 1.35 | 11.32 | Nov / Sep | 0.05 | 0.02 | 0.15 |
| Jun / Oct | 0.32 | 0.17 | 0.61 | Oct / Nov | *2.95 | 2.25 | 3.86 | Jul / Nov | *11.87 | 4.00 | 35.21 | Nov / Oct | 0.33 | 0.20 | 0.55 |
| Jun / Sep | 0.79 | 0.45 | 1.41 | Sep / Apr | *2.41 | 1.08 | 5.40 | Jul / Dec | 1.08 | 0.37 | 3.16 | Dec / Jan | *5.07 | 1.49 | 17.31 |
| Mar / May | 1.72 | 0.77 | 3.87 | Sep / Aug | 0.98 | 0.41 | 2.35 | Aug / Sep | 1.42 | 0.42 | 4.75 | Dec / Feb | 1.38 | 0.74 | 2.57 |
| Mar / Nov | 1.88 | 0.79 | 4.50 | Sep / Dec | 1.11 | 0.51 | 2.39 | Aug / Oct | *8.51 | 2.71 | 26.77 | Dec / Mar | 1.87 | 0.59 | 5.97 |
| Mar / Oct | 0.64 | 0.27 | 1.51 | Sep / Feb | 1.47 | 0.83 | 2.59 | Aug / Nov | *25.83 | 7.98 | 83.57 | Dec / Apr | *7.46 | 2.70 | 20.61 |
| Mar / Sep | 1.59 | 0.65 | 3.90 | Sep / Jan | 1.02 | 0.39 | 2.62 | Aug / Dec | 2.35 | 0.74 | 7.45 | Dec / May | 1.28 | 0.61 | 2.66 |
| May / Nov | 1.09 | 0.57 | 2.10 | Sep / Jul | 1.25 | 0.55 | 2.88 | Sep / Oct | *6.00 | 2.23 | 16.15 | Dec / Jun | *9.38 | 4.07 | 21.60 |
| May / Oct | 0.37 | 0.19 | 0.70 | Sep / Jun | 1.26 | 0.71 | 2.23 | Sep / Nov | *18.19 | 6.51 | 50.86 | Dec / Jul | 0.92 | 0.32 | 2.70 |
| May / Sep | 0.92 | 0.53 | 1.60 | Sep / Mar | 0.63 | 0.26 | 1.54 | Sep / Dec | 1.66 | 0.66 | 4.17 | Dec / Aug | 0.42 | 0.13 | 1.35 |
| Nov / Oct | 0.34 | 0.26 | 0.44 | Sep / May | 1.08 | 0.62 | 1.87 | Oct / Nov | *3.03 | 1.83 | 5.02 | Dec / Sep | 0.60 | 0.24 | 1.52 |
| Nov / Sep | 0.85 | 0.39 | 1.82 | Sep / Nov | 1.18 | 0.55 | 2.55 | Oct / Dec | 0.28 | 0.11 | 0.70 | Dec / Oct | *3.62 | 1.43 | 9.13 |
| Oct / Sep | *2.49 | 1.17 | 5.32 | Sep / Oct | 0.40 | 0.19 | 0.85 | Nov / Dec | 0.09 | 0.03 | 0.24 | Dec / Nov | *10.98 | 4.19 | 28.76 |

^1^Lower confidence limit

^2^Upper confidence limit * Significant pairwise comparisons

**Supplementary Table 6:** *Pairwise Odds Ratios (ORs) and 95% Confidence Intervals for Total Sperm Motility and Forward Progressive Sperm Motility in Litoria littlejohni, Comparing 12-Month Time Points (n = 10 per treatment).*

| **Total Sperm Motility** | | | | | | | | **Forward Progressive Sperm Motility** | | | | | | | |
| --- | --- | --- | --- | --- | --- | --- | --- | --- | --- | --- | --- | --- | --- | --- | --- |
| **Contrast** | **Ratio** | **LCL^1^** | **UCL^2^** | **Reverse Contrast** | **Ratio** | **LCL^1^** | **UCL^2^** | **Contrast** | **Ratio** | **LCL^1^** | **UCL^2^** | **Reverse Contrast** | **Ratio** | **LCL^1^** | **UCL^2^** |
| Apr / Aug | 0.54 | 0.21 | 1.40 | Aug / Apr | 1.86 | 0.71 | 4.83 | Jan / Feb | 1.91 | 0.49 | 7.41 | Feb / Jan | 0.52 | 0.13 | 2.02 |
| Apr / Dec | 0.41 | 0.18 | 0.95 | Dec / Apr | *2.42 | 1.05 | 5.58 | Jan / Mar | 1.73 | 0.37 | 8.04 | Mar / Jan | 0.58 | 0.12 | 2.67 |
| Apr / Feb | 0.71 | 0.31 | 1.62 | Dec / Aug | 1.30 | 0.53 | 3.21 | Jan / Apr | 1.40 | 0.33 | 5.87 | Mar / Feb | 1.10 | 0.34 | 3.57 |
| Apr / Jan | 1.03 | 0.33 | 3.23 | Feb / Apr | 1.41 | 0.62 | 3.23 | Jan / May | 3.14 | 0.82 | 12.06 | Apr / Jan | 0.72 | 0.17 | 3.01 |
| Apr / Jul | 0.37 | 0.15 | 0.94 | Feb / Aug | 0.76 | 0.31 | 1.86 | Jan / Jun | 0.98 | 0.25 | 3.81 | Apr / Feb | 1.37 | 0.49 | 3.85 |
| Apr / Jun | 1.28 | 0.57 | 2.85 | Feb / Dec | 0.58 | 0.36 | 0.94 | Jan / Jul | 0.36 | 0.08 | 1.55 | Apr / Mar | 1.24 | 0.35 | 4.35 |
| Apr / Mar | 0.47 | 0.17 | 1.27 | Jan / Apr | 0.97 | 0.31 | 3.02 | Jan / Aug | 0.50 | 0.11 | 2.24 | May / Jan | 0.32 | 0.08 | 1.23 |
| Apr / May | 0.40 | 0.18 | 0.88 | Jan / Aug | 0.52 | 0.16 | 1.71 | Jan / Sep | 0.84 | 0.20 | 3.61 | May / Feb | 0.61 | 0.30 | 1.25 |
| Apr / Nov | 0.45 | 0.19 | 1.07 | Jan / Dec | 0.40 | 0.13 | 1.19 | Jan / Oct | 2.24 | 0.56 | 8.92 | May / Mar | 0.55 | 0.18 | 1.70 |
| Apr / Oct | 1.44 | 0.63 | 3.29 | Jan / Feb | 0.68 | 0.23 | 2.03 | Jan / Nov | 0.65 | 0.16 | 2.64 | May / Apr | 0.45 | 0.17 | 1.19 |
| Apr / Sep | 0.09 | 0.04 | 0.23 | Jul / Apr | *2.68 | 1.06 | 6.78 | Jan / Dec | 0.22 | 0.05 | 0.87 | Jun / Jan | 1.02 | 0.26 | 3.95 |
| Aug / Dec | 0.77 | 0.31 | 1.89 | Jul / Aug | 1.44 | 0.54 | 3.88 | Feb / Mar | 0.91 | 0.28 | 2.93 | Jun / Feb | 1.95 | 0.90 | 4.23 |
| Aug / Feb | 1.32 | 0.54 | 3.23 | Jul / Dec | 1.11 | 0.46 | 2.65 | Feb / Apr | 0.73 | 0.26 | 2.05 | Jun / Mar | 1.77 | 0.56 | 5.53 |
| Aug / Jan | 1.92 | 0.59 | 6.31 | Jul / Feb | 1.90 | 0.80 | 4.52 | Feb / May | 1.64 | 0.80 | 3.36 | Jun / Apr | 1.42 | 0.52 | 3.86 |
| Aug / Jul | 0.69 | 0.26 | 1.86 | Jul / Jan | 2.78 | 0.87 | 8.91 | Feb / Jun | 0.51 | 0.24 | 1.11 | Jun / May | *3.20 | 2.14 | 4.76 |
| Aug / Jun | 2.38 | 0.99 | 5.67 | Jun / Apr | 0.78 | 0.35 | 1.74 | Feb / Jul | 0.19 | 0.06 | 0.55 | Jul / Jan | 2.78 | 0.64 | 11.98 |
| Aug / Mar | 0.87 | 0.30 | 2.50 | Jun / Aug | 0.42 | 0.18 | 1.01 | Feb / Aug | 0.26 | 0.09 | 0.78 | Jul / Feb | *5.31 | 1.83 | 15.43 |
| Aug / May | 0.74 | 0.31 | 1.75 | Jun / Dec | 0.32 | 0.16 | 0.64 | Feb / Sep | 0.44 | 0.20 | 0.98 | Jul / Mar | *4.82 | 1.34 | 17.35 |
| Aug / Nov | 0.84 | 0.34 | 2.12 | Jun / Feb | 0.55 | 0.29 | 1.07 | Feb / Oct | 1.17 | 0.47 | 2.93 | Jul / Apr | *3.88 | 1.23 | 12.27 |
| Aug / Oct | *2.68 | 1.09 | 6.55 | Jun / Jan | 0.81 | 0.28 | 2.35 | Feb / Nov | 0.34 | 0.13 | 0.87 | Jul / May | *8.71 | 3.16 | 24.01 |
| Aug / Sep | 0.18 | 0.07 | 0.46 | Jun / Jul | 0.29 | 0.13 | 0.68 | Feb / Dec | 0.11 | 0.05 | 0.25 | Jul / Jun | 2.73 | 0.97 | 7.64 |
| Dec / Feb | *1.72 | 1.06 | 2.78 | Mar / Apr | 2.14 | 0.79 | 5.81 | Mar / Apr | 0.81 | 0.23 | 2.82 | Aug / Jan | 1.99 | 0.45 | 8.87 |
| Dec / Jan | 2.51 | 0.84 | 7.48 | Mar / Aug | 1.15 | 0.40 | 3.31 | Mar / May | 1.81 | 0.59 | 5.57 | Aug / Feb | *3.81 | 1.28 | 11.36 |
| Dec / Jul | 0.90 | 0.38 | 2.16 | Mar / Dec | 0.88 | 0.34 | 2.28 | Mar / Jun | 0.57 | 0.18 | 1.77 | Aug / Mar | 3.46 | 0.92 | 12.97 |
| Dec / Jun | *3.10 | 1.55 | 6.19 | Mar / Feb | 1.51 | 0.59 | 3.89 | Mar / Jul | 0.21 | 0.06 | 0.75 | Aug / Apr | 2.78 | 0.84 | 9.18 |
| Dec / Mar | 1.13 | 0.44 | 2.93 | Mar / Jan | 2.21 | 0.65 | 7.53 | Mar / Aug | 0.29 | 0.08 | 1.09 | Aug / May | *6.25 | 2.14 | 18.28 |
| Dec / May | 0.97 | 0.50 | 1.88 | Mar / Jul | 0.80 | 0.28 | 2.24 | Mar / Sep | 0.48 | 0.14 | 1.70 | Aug / Jun | 1.96 | 0.66 | 5.80 |
| Dec / Nov | 1.10 | 0.50 | 2.44 | Mar / Jun | 2.73 | 1.09 | 6.86 | Mar / Oct | 1.29 | 0.39 | 4.24 | Aug / Jul | 0.72 | 0.21 | 2.43 |
| Dec / Oct | *3.49 | 1.63 | 7.49 | May / Apr | *2.51 | 1.14 | 5.49 | Mar / Nov | 0.38 | 0.11 | 1.26 | Sep / Jan | 1.19 | 0.28 | 5.12 |
| Dec / Sep | 0.23 | 0.10 | 0.51 | May / Aug | 1.35 | 0.57 | 3.18 | Mar / Dec | 0.13 | 0.04 | 0.44 | Sep / Feb | *2.28 | 1.02 | 5.08 |
| Feb / Jan | 1.46 | 0.49 | 4.34 | May / Dec | 1.03 | 0.53 | 2.01 | Apr / May | 2.25 | 0.84 | 5.98 | Sep / Mar | 2.07 | 0.59 | 7.28 |
| Feb / Jul | 0.53 | 0.22 | 1.25 | May / Feb | 1.77 | 0.95 | 3.30 | Apr / Jun | 0.70 | 0.26 | 1.91 | Sep / Apr | 1.66 | 0.54 | 5.16 |
| Feb / Jun | 1.80 | 0.94 | 3.48 | May / Jan | 2.59 | 0.90 | 7.45 | Apr / Jul | 0.26 | 0.08 | 0.81 | Sep / May | *3.74 | 1.76 | 7.91 |
| Feb / Mar | 0.66 | 0.26 | 1.70 | May / Jul | 0.93 | 0.41 | 2.13 | Apr / Aug | 0.36 | 0.11 | 1.19 | Sep / Jun | 1.17 | 0.51 | 2.66 |
| Feb / May | 0.56 | 0.30 | 1.05 | May / Jun | *3.20 | 2.22 | 4.61 | Apr / Sep | 0.60 | 0.19 | 1.87 | Sep / Jul | 0.43 | 0.13 | 1.37 |
| Feb / Nov | 0.64 | 0.29 | 1.41 | May / Mar | 1.17 | 0.47 | 2.90 | Apr / Oct | 1.60 | 0.57 | 4.55 | Sep / Aug | 0.60 | 0.18 | 2.02 |
| Feb / Oct | 2.03 | 0.95 | 4.33 | Nov / Apr | 2.20 | 0.94 | 5.19 | Apr / Nov | 0.47 | 0.16 | 1.35 | Oct / Jan | 0.45 | 0.11 | 1.78 |
| Feb / Sep | 0.13 | 0.06 | 0.29 | Nov / Aug | 1.19 | 0.47 | 2.98 | Apr / Dec | 0.16 | 0.05 | 0.47 | Oct / Feb | 0.85 | 0.34 | 2.13 |
| Jan / Jul | 0.36 | 0.11 | 1.16 | Nov / Dec | 0.91 | 0.41 | 2.01 | May / Jun | 0.31 | 0.21 | 0.47 | Oct / Mar | 0.77 | 0.24 | 2.54 |
| Jan / Jun | 1.24 | 0.42 | 3.59 | Nov / Feb | 1.56 | 0.71 | 3.44 | May / Jul | 0.11 | 0.04 | 0.32 | Oct / Apr | 0.62 | 0.22 | 1.77 |
| Jan / Mar | 0.45 | 0.13 | 1.54 | Nov / Jan | 2.28 | 0.75 | 6.91 | May / Aug | 0.16 | 0.05 | 0.47 | Oct / May | 1.40 | 0.58 | 3.40 |
| Jan / May | 0.39 | 0.13 | 1.11 | Nov / Jul | 0.82 | 0.34 | 2.00 | May / Sep | 0.27 | 0.13 | 0.57 | Oct / Jun | 0.44 | 0.18 | 1.08 |
| Jan / Nov | 0.44 | 0.14 | 1.33 | Nov / Jun | *2.82 | 1.31 | 6.03 | May / Oct | 0.71 | 0.29 | 1.73 | Oct / Jul | 0.16 | 0.05 | 0.47 |
| Jan / Oct | 1.39 | 0.47 | 4.13 | Nov / Mar | 1.03 | 0.39 | 2.71 | May / Nov | 0.21 | 0.08 | 0.52 | Oct / Aug | 0.22 | 0.07 | 0.68 |
| Jan / Sep | 0.09 | 0.03 | 0.29 | Nov / May | 0.88 | 0.42 | 1.85 | May / Dec | 0.07 | 0.03 | 0.17 | Oct / Sep | 0.37 | 0.13 | 1.07 |
| Jul / Jun | *3.43 | 1.48 | 7.94 | Oct / Apr | 0.69 | 0.30 | 1.59 | Jun / Jul | 0.37 | 0.13 | 1.03 | Nov / Jan | 1.53 | 0.38 | 6.18 |
| Jul / Mar | 1.26 | 0.45 | 3.52 | Oct / Aug | 0.37 | 0.15 | 0.91 | Jun / Aug | 0.51 | 0.17 | 1.52 | Nov / Feb | *2.93 | 1.15 | 7.45 |
| Jul / May | 1.07 | 0.47 | 2.44 | Oct / Dec | 0.29 | 0.13 | 0.61 | Jun / Sep | 0.86 | 0.38 | 1.95 | Nov / Mar | 2.65 | 0.79 | 8.85 |
| Jul / Nov | 1.22 | 0.50 | 2.98 | Oct / Feb | 0.49 | 0.23 | 1.05 | Jun / Oct | 2.28 | 0.92 | 5.65 | Nov / Apr | 2.14 | 0.74 | 6.16 |
| Jul / Oct | *3.87 | 1.63 | 9.18 | Oct / Jan | 0.72 | 0.24 | 2.13 | Jun / Nov | 0.67 | 0.26 | 1.68 | Nov / May | *4.80 | 1.94 | 11.86 |
| Jul / Sep | 0.25 | 0.10 | 0.64 | Oct / Jul | 0.26 | 0.11 | 0.61 | Jun / Dec | 0.22 | 0.09 | 0.57 | Nov / Jun | 1.50 | 0.59 | 3.79 |
| Jun / Mar | 0.37 | 0.15 | 0.92 | Oct / Jun | 0.89 | 0.43 | 1.83 | Jul / Aug | 1.39 | 0.41 | 4.73 | Nov / Jul | 0.55 | 0.19 | 1.64 |
| Jun / May | 0.31 | 0.22 | 0.45 | Oct / Mar | 0.32 | 0.13 | 0.83 | Jul / Sep | 2.33 | 0.73 | 7.46 | Nov / Aug | 0.77 | 0.25 | 2.38 |
| Jun / Nov | 0.36 | 0.17 | 0.76 | Oct / May | 0.28 | 0.14 | 0.56 | Jul / Oct | *6.22 | 2.13 | 18.18 | Nov / Sep | 1.28 | 0.44 | 3.72 |
| Jun / Oct | 1.13 | 0.55 | 2.33 | Oct / Nov | 0.32 | 0.20 | 0.50 | Jul / Nov | 1.82 | 0.61 | 5.40 | Nov / Oct | *3.43 | 2.31 | 5.08 |
| Jun / Sep | 0.07 | 0.04 | 0.14 | Sep / Apr | *10.60 | 4.30 | 26.12 | Jul / Dec | 0.61 | 0.20 | 1.89 | Dec / Jan | *4.57 | 1.14 | 18.22 |
| Mar / May | 0.85 | 0.34 | 2.11 | Sep / Aug | *5.71 | 2.18 | 14.97 | Aug / Sep | 1.67 | 0.50 | 5.64 | Dec / Feb | *8.73 | 3.95 | 19.30 |
| Mar / Nov | 0.97 | 0.37 | 2.56 | Sep / Dec | *4.37 | 1.94 | 9.85 | Aug / Oct | *4.46 | 1.46 | 13.60 | Dec / Mar | *7.92 | 2.29 | 27.32 |
| Mar / Oct | *3.08 | 1.20 | 7.90 | Sep / Feb | *7.51 | 3.42 | 16.48 | Aug / Nov | 1.30 | 0.42 | 4.04 | Dec / Apr | *6.38 | 2.11 | 19.28 |
| Mar / Sep | 0.20 | 0.07 | 0.55 | Sep / Jan | *10.97 | 3.49 | 34.45 | Aug / Dec | 0.44 | 0.14 | 1.41 | Dec / May | *14.32 | 5.73 | 35.76 |
| May / Nov | 1.14 | 0.54 | 2.39 | Sep / Jul | *3.95 | 1.55 | 10.06 | Sep / Oct | 2.67 | 0.94 | 7.61 | Dec / Jun | *4.48 | 1.75 | 11.51 |
| May / Oct | *3.61 | 1.77 | 7.35 | Sep / Jun | *13.55 | 7.13 | 25.76 | Sep / Nov | 0.78 | 0.27 | 2.26 | Dec / Jul | 1.64 | 0.53 | 5.12 |
| May / Sep | 0.24 | 0.14 | 0.41 | Sep / Mar | *4.96 | 1.81 | 13.59 | Sep / Dec | 0.26 | 0.08 | 0.82 | Dec / Aug | 2.29 | 0.71 | 7.38 |
| Nov / Oct | *3.17 | 2.00 | 5.02 | Sep / May | *4.23 | 2.46 | 7.28 | Oct / Nov | 0.29 | 0.20 | 0.43 | Dec / Sep | *3.83 | 1.21 | 12.11 |
| Nov / Sep | 0.21 | 0.09 | 0.49 | Sep / Nov | *4.81 | 2.03 | 11.43 | Oct / Dec | 0.10 | 0.03 | 0.27 | Dec / Oct | *10.23 | 3.66 | 28.59 |
| Oct / Sep | 0.07 | 0.03 | 0.15 | Sep / Oct | *15.27 | 6.61 | 35.30 | Nov / Dec | 0.33 | 0.12 | 0.96 | Dec / Nov | *2.99 | 1.05 | 8.52 |

^1^Lower confidence limit

^2^Upper confidence limit * Significant pairwise comparisons

**Supplementary Table 7:** *Pairwise Incidence Rate Ratios (IRRs) and 95% Confidence Intervals for Sperm Concentration in Litoria littlejohni, Comparing Month and Wild vs. Captive Conditions Across Four Months (n = 3–11 per treatment).*

| **Contrast** | **Ratio** | **LCL^1^** | **UCL^2^** | **Reverse Contrast** | **Ratio** | **LCL^1^** | **UCL^2^** |
| --- | --- | --- | --- | --- | --- | --- | --- |
| Jun Captive / Jul Captive | 1.08 | 0.65 | 1.77 | Jul Captive / Jun Captive | 0.93 | 0.56 | 1.53 |
| Jun Captive / Aug Captive | 0.83 | 0.49 | 1.39 | Aug Captive / Jun Captive | 1.21 | 0.72 | 2.04 |
| Jun Captive / Sep Captive | 1.16 | 0.61 | 2.21 | Aug Captive / Jul Captive | 1.30 | 0.77 | 2.20 |
| Jun Captive / Jun Wild | 0.35 | 0.16 | 0.76 | Sep Captive / Jun Captive | 0.86 | 0.45 | 1.64 |
| Jun Captive / Jul Wild | 0.54 | 0.24 | 1.19 | Sep Captive / Jul Captive | 0.93 | 0.49 | 1.76 |
| Jun Captive / Aug Wild | *3.71 | 1.29 | 10.67 | Sep Captive / Aug Captive | 0.71 | 0.37 | 1.36 |
| Jun Captive / Sep Wild | 0.70 | 0.22 | 2.24 | Jun Wild / Jun Captive | *2.90 | 1.32 | 6.35 |
| Jul Captive / Aug Captive | 0.77 | 0.45 | 1.30 | Jun Wild / Jul Captive | *3.12 | 1.42 | 6.84 |
| Jul Captive / Sep Captive | 1.08 | 0.57 | 2.05 | Jun Wild / Aug Captive | *2.39 | 1.07 | 5.34 |
| Jul Captive / Jun Wild | 0.32 | 0.15 | 0.70 | Jun Wild / Sep Captive | *3.36 | 1.38 | 8.16 |
| Jul Captive / Jul Wild | 0.50 | 0.22 | 1.11 | Jul Wild / Jun Captive | 1.87 | 0.84 | 4.16 |
| Jul Captive / Aug Wild | *3.45 | 1.20 | 9.92 | Jul Wild / Jul Captive | 2.01 | 0.90 | 4.48 |
| Jul Captive / Sep Wild | 0.65 | 0.20 | 2.08 | Jul Wild / Aug Captive | 1.54 | 0.68 | 3.48 |
| Aug Captive / Sep Captive | 1.41 | 0.73 | 2.69 | Jul Wild / Sep Captive | 2.17 | 0.89 | 5.29 |
| Aug Captive / Jun Wild | 0.42 | 0.19 | 0.93 | Jul Wild / Jun Wild | 0.64 | 0.34 | 1.21 |
| Aug Captive / Jul Wild | 0.65 | 0.29 | 1.46 | Aug Wild / Jun Captive | 0.27 | 0.09 | 0.78 |
| Aug Captive / Aug Wild | *4.49 | 1.54 | 13.08 | Aug Wild / Jul Captive | 0.29 | 0.10 | 0.84 |
| Aug Captive / Sep Wild | 0.85 | 0.26 | 2.74 | Aug Wild / Aug Captive | 0.22 | 0.08 | 0.65 |
| Sep Captive / Jun Wild | 0.30 | 0.12 | 0.72 | Aug Wild / Sep Captive | 0.31 | 0.10 | 0.97 |
| Sep Captive / Jul Wild | 0.46 | 0.19 | 1.13 | Aug Wild / Jun Wild | 0.09 | 0.03 | 0.25 |
| Sep Captive / Aug Wild | *3.20 | 1.03 | 9.91 | Aug Wild / Jul Wild | 0.14 | 0.05 | 0.40 |
| Sep Captive / Sep Wild | 0.60 | 0.18 | 2.06 | Sep Wild / Jun Captive | 1.43 | 0.45 | 4.59 |
| Jun Wild / Jul Wild | 1.55 | 0.83 | 2.91 | Sep Wild / Jul Captive | 1.54 | 0.48 | 4.94 |
| Jun Wild / Aug Wild | *10.74 | 3.96 | 29.11 | Sep Wild / Aug Captive | 1.18 | 0.37 | 3.83 |
| Jun Wild / Sep Wild | 2.02 | 0.67 | 6.14 | Sep Wild / Sep Captive | 1.66 | 0.48 | 5.70 |
| Jul Wild / Aug Wild | *6.93 | 2.53 | 18.99 | Sep Wild / Jun Wild | 0.49 | 0.16 | 1.50 |
| Jul Wild / Sep Wild | 1.30 | 0.42 | 4.00 | Sep Wild / Jul Wild | 0.77 | 0.25 | 2.35 |
| Aug Wild / Sep Wild | 0.19 | 0.05 | 0.70 | Sep Wild / Aug Wild | *5.31 | 1.42 | 19.82 |

^1^Lower confidence limit

^2^Upper confidence limit

- Significant pairwise comparisons

**Supplementary Table 8:** *Pairwise Odds Ratios (ORs) and 95% Confidence Intervals for Sperm Membrane Integrity in Litoria littlejohni, Comparing Month and Wild vs. Captive Conditions Across Four Months (n = 3–11 per treatment).*

| **Contrast** | **Ratio** | **LCL^1^** | **UCL^2^** | **Reverse Contrast** | **Ratio** | **LCL^1^** | **UCL^2^** |
| --- | --- | --- | --- | --- | --- | --- | --- |
| Jun / Jul | 0.51 | 0.26 | 0.98 | Jul / Jun | *1.97 | 1.02 | 3.78 |
| Jun / Aug | 0.30 | 0.13 | 0.68 | Aug / Jun | *3.31 | 1.47 | 7.45 |
| Jun / Sep | 1.03 | 0.43 | 2.49 | Aug / Jul | 1.68 | 0.75 | 3.77 |
| Jul / Aug | 0.59 | 0.27 | 1.33 | Sep / Jun | 0.97 | 0.40 | 2.33 |
| Jul / Sep | 2.03 | 0.83 | 4.96 | Sep / Jul | 0.49 | 0.20 | 1.20 |
| Aug / Sep | *3.42 | 1.26 | 9.29 | Sep / Aug | 0.29 | 0.11 | 0.79 |

^1^Lower confidence limit

^2^Upper confidence limit

- Significant pairwise comparisons

**Supplementary Table 9:** *Pairwise Odds Ratios (ORs) and 95% Confidence Intervals for Sperm Total Motility in Litoria littlejohni, Comparing Month and Wild vs. Captive Conditions Across Four Months (n = 3–11 per treatment).*

| **Contrast** | **Ratio** | **LCL^1^** | **UCL^2^** | **Reverse Contrast** | **Ratio** | **LCL^1^** | **UCL^2^** |
| --- | --- | --- | --- | --- | --- | --- | --- |
| Jun / Jul | 0.57 | 0.36 | 0.90 | Jul / Jun | *1.76 | 1.12 | 2.76 |
| Jun / Aug | 1.03 | 0.61 | 1.75 | Aug / Jun | 0.97 | 0.57 | 1.63 |
| Jun / Sep | 0.52 | 0.28 | 0.99 | Aug / Jul | 0.55 | 0.32 | 0.94 |
| Jul / Aug | *1.82 | 1.07 | 3.10 | Sep / Jun | *1.90 | 1.01 | 3.59 |
| Jul / Sep | 0.92 | 0.49 | 1.75 | Sep / Jul | 1.08 | 0.57 | 2.06 |
| Aug / Sep | 0.51 | 0.26 | 1.00 | Sep / Aug | *1.97 | 1.00 | 3.87 |

^1^Lower confidence limit

^2^Upper confidence limit

- Significant pairwise comparisons
